# Preferential expression of scores of functionally and evolutionarily diverse DNA and RNA-binding proteins during *Oxytricha trifallax* macronuclear development

**DOI:** 10.1371/journal.pone.0170870

**Published:** 2017-02-16

**Authors:** Zachary T. Neeb, Daniel J. Hogan, Sol Katzman, Alan M. Zahler

**Affiliations:** 1 Department of Molecular, Cell and Developmental Biology and Center for Molecular Biology of RNA, University of California Santa Cruz, Santa Cruz, California, United States of America; 2 Tocagen Inc., San Diego, California, United States of America; 3 Center for Biomolecular Science and Engineering, University of California Santa Cruz, Santa Cruz, California, United States of America; International Centre for Genetic Engineering and Biotechnology, ITALY

## Abstract

During its sexual reproduction, the stichotrichous ciliate *Oxytricha trifallax* orchestrates a remarkable transformation of one of the newly formed germline micronuclear genomes. Hundreds of thousands of gene pieces are stitched together, excised from chromosomes, and replicated dozens of times to yield a functional somatic macronuclear genome composed of ~16,000 distinct DNA molecules that typically encode a single gene. Little is known about the proteins that carry out this process. We profiled mRNA expression as a function of macronuclear development and identified hundreds of mRNAs preferentially expressed at specific times during the program. We find that a disproportionate number of these mRNAs encode proteins that are involved in DNA and RNA functions. Many mRNAs preferentially expressed during macronuclear development have paralogs that are either expressed constitutively or are expressed at different times during macronuclear development, including many components of the RNA polymerase II machinery and homologous recombination complexes. Hundreds of macronuclear development-specific genes encode proteins that are well-conserved among multicellular eukaryotes, including many with links to germline functions or development. Our work implicates dozens of DNA and RNA-binding proteins with diverse evolutionary trajectories in macronuclear development in *O*. *trifallax*. It suggests functional connections between the process of macronuclear development in unicellular ciliates and germline specialization and differentiation in multicellular organisms, and argues that gene duplication is a key source of evolutionary innovation in this process.

## Introduction

Ciliates are diverse, abundant and extremely successful unicellular eukaryotes that display a special case of germline-soma specialization vis-à-vis nuclear dimorphism; a germline nucleus (micronucleus) used for propagation of genetic information and a somatic nucleus (macronucleus) used for cell growth [1]. When starved, cells of different mating types pair, micronuclei undergo meiosis, they exchange haploid micronuclei which fuse to form a new diploid micronucleus, they perform one to several rounds of micronuclear mitosis, and then develop a new macronucleus from one of the newly formed micronuclei. This differentiation program is associated with reorganization of the genome and in some cases extraordinary genome rearrangements. In all ciliate lineages studied, genome rearrangements are epigenetically determined by communication of DNA content between macronuclei and micronuclei via RNA intermediates [2–4]. Several factors intrinsic to germline specialization and differentiation were first characterized in ciliates, such as telomerase [5] and histone acetyl transferase [6], as well as seminal studies on specialized histones, PIWI and HP1 proteins [7–19]. Thus, ciliates provide relatively simple and facile systems to study principles of germline-soma specialization, germline differentiation and RNA-mediated epigenetic memory.

Stichotrichous ciliates are a special case in which nuclear duality led to the evolution of two extraordinary and distinct genomes [20]. While micronuclear DNA is organized on long chromosomes similar to other eukaryotes, genes are interrupted by multiple, short, noncoding DNA sequences called internally eliminated sequences (IESs) that interrupt gene pieces, called macronuclear destined sequences (MDSs) [21]. During macronuclear development IESs are precisely eliminated and MDSs are recombined to form a functional gene [1]. In some genes, MDSs are in a scrambled disorder. MDS recombination must also ensure that scrambled gene pieces are precisely unscrambled in order to form functional macronuclear genes [22]. Some genes are scrambled into more than 50 MDSs, sometimes dispersed over multiple loci [23]. There are >200,000 IESs and >3,000 scrambled genes in *Oxytricha trifallax*, the only stichotrich whose micronuclear genome has been sequenced [24].

The macronuclear DNA molecules of stichotrichous ciliates, averaging ~2kb in length, are the smallest known in nature [25]. Each DNA molecule, present at 100–100,000 copies per macronucleus, typically contains a single coding sequence along with regulatory information and short telomeres [7, 26–28]. At the onset of macronuclear development, micronuclear chromosomes undergo polytenization. Subsequently, MDSs are unscrambled and spliced while IESs, non-genic DNA and transposable elements are removed and degraded, and gene-sized molecules are excised from chromosomes. Telomeres are added *de novo* and these small DNA molecules are then replicated dozens of times and to form a mature, functional macronucleus (S1 Fig) (For reviews of macronuclear development, see [20, 29–31]). During this process there are millions of precise DNA splicing and ligation events resulting in a streamlined somatic genome of 20-fold reduced complexity. The diversity and sheer magnitude of DNA splicing and processing in stichotrichs dwarf those in the better characterized and distantly related ciliates *Tetrahymena* and *Paramecium*.

While these phenomena were first described over twenty-five years ago [21, 22, 32, 33], we are now just beginning to characterize the molecular mechanisms underlying this extraordinary genome transformation. A key question surrounds how specific DNA sequences are precisely recombined, retained and amplified while others are excised and eliminated. For instance, the junctions of MDSs and IESs contain short “pointer” sequences that are likely involved, but inadequate, for proper MDS splicing, as their sequence can occur multiple times within the gene [34]. Therefore, building on studies in *Paramecium*, David Prescott proposed that a template DNA or RNA from the parental macronucleus must guide MDS splicing [3, 35, 36]. Long dsRNAs, corresponding to entire macronuclear DNA molecules, are produced early during macronuclear development and are suggested to act as the proposed "templates" [37]. Injection of synthetic long dsRNAs with altered MDS arrangements led to production of correspondingly altered macronuclear DNA molecules, not only in the injected cells, but in offspring as well, suggesting non-Mendelian inheritance through these RNA templates. In addition, 27 nucleotide small RNAs mapping to both strands of macronuclear DNA molecules, called 27macRNAs, are produced *en masse* during early macronuclear development [38, 39]. These 27macRNAs are associated with a PIWI homolog called Otiwi1, and are also referred to as piRNAs. This class of small RNAs specify which segments of micronuclear DNA will be protected from degradation during macronuclear development [39], perhaps by specifying DNA methylation of MDSs [40]. The relationship between 27mer piRNAs and the long dsRNA “templates” involved in MDS rearrangements remains unknown.

Correspondingly little is known about the protein machinery involved in genome conversion in strichotrichs. Electron microscopy studies show dramatic reorganization of chromatin and nuclear architecture during the developmental program [41–44]. Analyses of single genes suggest extensive chromatin changes occur during macronuclear development and that chromatin marks distinguish DNA regions with different fates [1, 40, 45, 46]. Not surprisingly stichotrichs encode a large array of histone proteins, several of which are expressed exclusively during macronuclear development [7, 11, 12]. The first study to identify mRNAs differentially expressed during macronuclear development, in *Stylonychia lemnae*, utilized subtractive cDNA hybridization and cloning [13]. This work identified Otiwi1, a protein containing the Alba nucleic acid binding domain, a novel Kelch domain protein, and several well-conserved DNA and RNA binding proteins [13, 47]. Another study suggested that the transposase encoded within a transposon family that is precisely excised during macronuclear development, called TBE transposons, is the enzyme responsible for producing dsDNA breaks for IES excision and MDS ligation [48]. Indeed “domesticated” transposases are implicated in IES excision in other ciliate lineages [49, 50]. It was recently reported that a paralog of RNA polymerase II second largest subunit is expressed exclusively during macronuclear development, and this factor, RPB2b, binds dsRNA templates first in the parental macronucleus and then in the developing macronucleus, suggesting a role in DNA rearrangements [51]. Electron microscopy studies from the 1970’s showed that, coincident with the mass reduction in DNA content, proteinaceous “vesicles” transect the polytene chromosomes; potentially these “vesicles” contain the protein machinery involved in excision of macronuclear destined DNA molecules and/or degradation of the rest of the genome [41, 52–54].

As an important step towards obtaining a system-level understanding of the developmental program in strichotrichs and identifying the molecular machinery involved, we characterized mRNA expression during macronuclear development in *O*. *trifallax* via high throughput sequencing. Our studies offer a unique insight into how *Oxytricha* simultaneously preserves and protects its germline nucleus, the new micronucleus, while also activating a somatic nucleus, the new macronucleus, through extensive DNA rearrangements and elimination.

## Results

### mRNA expression profiles of macronuclear development in *Oxytricha trifallax*

In order to identify mRNAs preferentially expressed during macronuclear development, we performed high-throughput sequencing of poly-A selected RNA isolated from seven time points; cultures growing vegetatively as well as 0, 6, 12, 24, 48 and 72 hours post-mixing of cells of complementary mating types, with biological replicates of each sample. Samples were sequenced to an average depth of 22 million paired-end reads. Raw sequencing reads were mapped to annotated macronuclear genome RNAs [26] using Tophat2 and normalized expression data in the form of fragments per kilobase million (FPKM) values were obtained with Cuffdiff2 [55, 56]. We obtained quality measurements (FPKM ≥ 3 in at least one experiment) from 17055 of 24885 annotated mRNAs (S1 Dataset).

We used several approaches to define gene function as manually curated information is available for only a handful of genes. Predicted protein domains and Gene Ontology (GO) terms for each gene were extracted from the *Oxytricha* genome database. We used Orthofinder to infer orthogroups (set of genes that are descended from a single gene in the last common ancestor of all the species being considered) among *O*. *trifallax*, stichotrichous ciliate *S*. *lemnae*, the distantly related ciliates *Tetrahymena thermophila* and *Paramecium tetraurelia*, as well as “model” organisms *Arabidopsis thaliana*, *Saccharomyces cerevisiae*, *Drosophila melanogaster*, *Caenorhabditis elegans* and human (S2 Dataset) [57]. We utilized annotations and published work for genes in the same orthogroup, protein domain information and previously published results to name and annotate 2300 *O*. *trifallax* genes, including 402 genes preferentially expressed during macronuclear development as defined below (S3 Dataset). As we expected many genes involved in macronuclear development to be linked to RNA- and DNA-related processes we also manually curated gene sets linked to RNA binding (“RNA”), DNA synthesis and repair (“DNA”), “chromatin” and RNA polymerase II mediated “transcription”.

**Fig 1:**
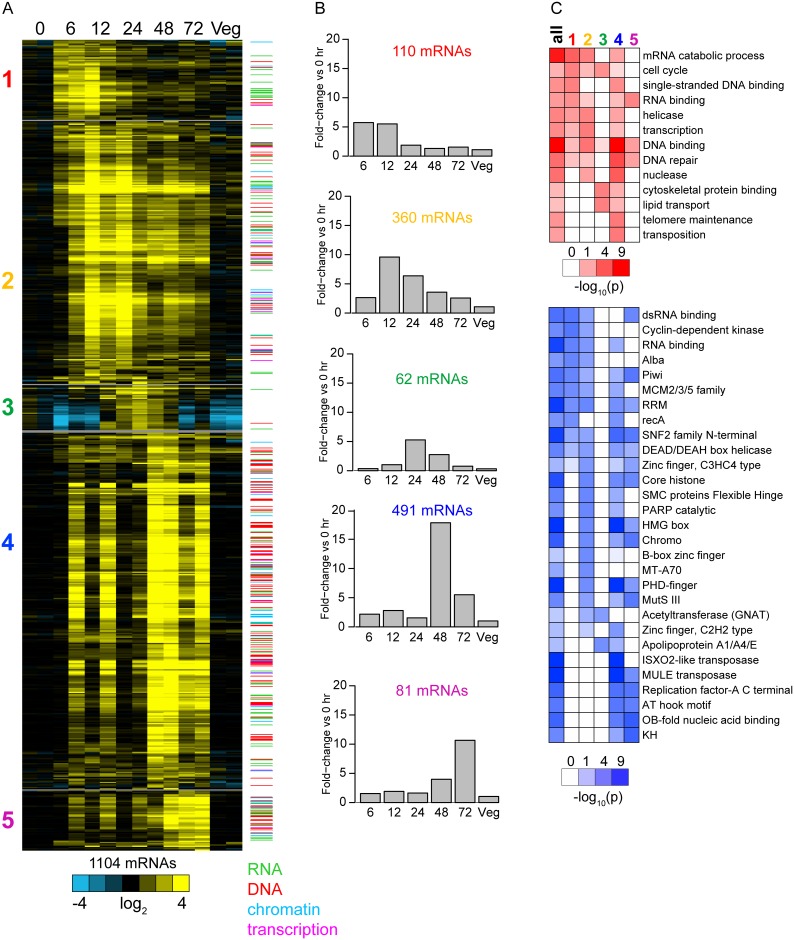
mRNA expression profiles of macronuclear development in *O*. *trifallax*. Heatmap representation of relative mRNA expression of 1104 mRNAs preferentially expressed during macronuclear development. mRNAs are grouped according to co-expression module (1–5) and within each module organized by hierarchical clustering. Relative mRNA expression was normalized such that log_2_(FPKM +1) levels in 0 hr cells was zero on average. The color bar to the right of the Fig indicates if the mRNA was in one of four manually curated gene sets linked to DNA and RNA biology.Barplot representation of the average relative expression of mRNAs in each module across the developmental program.(top) GO terms (rows) associated with macronuclear development-specific mRNAs and mRNAs in modules 1–5 (columns). The significance of enrichment of the GO term is represented as a heat map (scale is below the figure) in which the color intensity corresponds to the negative log_10_ p-value. Only a subset of significantly enriched GO terms are shown. (bottom) Same as top except for protein domains.

To visualize mRNA expression as a function of macronuclear development, FPKM values were log_2_ +1 transformed and the average value from the zero hour time point was subtracted from each sample. mRNAs preferentially expressed during macronuclear development were defined as those in which the average fold change for one or more developmental time points was at least 3-fold greater than 0 hour and vegetative cells, resulting in 1162 mRNAs. We organized the 1162 mRNAs according to their relative expression during macronuclear development using weighted correlation network analysis (WGCNA)[58], which identified six expression modules (Fig 1A), corresponding to different temporal patterns (Fig 1B). One of the modules, including 58 mRNAs, was omitted because the mRNAs were abundantly expressed in one of two samples from vegetative cells and thus likely not specific for macronuclear development (S4 Dataset).

We utilized the compiled annotations to glean broad themes among mRNAs preferentially expressed during macronuclear development. Relative to all mRNAs for which we obtained quality measurements, there was a striking enrichment for mRNAs encoding proteins linked to DNA and RNA metabolism (Fig 1C); for instance, 105 of 614 mRNAs that are annotated as “DNA binding” among mRNAs with quality measurements were preferentially expressed during macronuclear development (hypergeometric density distribution, p < 1e-16). In that same regard, 19 of 84 “DNA replication” (p < 1e-4), 24 of 155 “DNA repair” (p = 0.001), and seven of eight “mRNA catabolic process” (p < 1e-7) mRNAs were preferentially expressed during macronuclear development. Correspondingly, among protein domains we observed an abundance related to RNA and DNA binding that were preferentially expressed during macronuclear development, including 28 of 106 RNA-recognition motif (RRM) (p <1e-8), four of five double-stranded RNA-binding domain (dsRBD) (p <1e-4), 19 of 31 high mobility group (HMG) box (p < 1e-13), 28 of 70 PHD-finger (p < 1e-13), 12 of 27 histones (p < 1e-6), seven of 25 Poly(ADP-ribose) polymerase (PARP) catalytic domain (p = 0.002), seven of 11 Chromo (p < 1e-5), four of 11 Alba (p = 0.006), ten of ten ISXO2-like transposase, ten of ten MULE transposase, nine of 20 OB-fold (p < 1e-5), 15 of 74 DEAD/DEAH box helicase (p = 0.0005), six of nine Replication factor-A C terminal (p < 1e-5) and four of six RAD51 (p = 0.0002). Using our manually curated lists, 107 of 987 RNA, 150 of 398 DNA, 61 of 365 chromatin and 25 of 113 transcription annotated genes were preferentially expressed during macronuclear development. There was a modest difference in the proportion of RNA and transcription mRNAs in the modules whose expression peaked earlier during macronuclear development (modules 1 and 2) relative to DNA and chromatin mRNAs (S2 Fig).

### Gene duplication and functional specialization of proteins involved in RNA and DNA metabolism

The primary goal of this work is to identify potential functions of mRNAs that may be involved in the DNA manipulations during macronuclear development. A key component of this is to characterize the evolutionary trajectory of these proteins. In the process of analyzing the mRNA expression data we noticed that many mRNAs whose expression was specific to macronuclear development appeared to have paralogs either not preferentially expressed during macronuclear development or expressed at a different time during macronuclear development. Gene duplication followed by divergence of mRNA expression is an indication of functional specialization and could be one mechanism by which *O*. *trifallax* acquired the machinery necessary to carry out the macronuclear development program [59]. Many of the previously characterized macronuclear development-specific genes are part of evolutionarily conserved families with multiple members in *O*. *trifallax* that have divergent expression patterns. For instance, *O*. *trifallax* encodes thirteen PIWI proteins, seven of which are macronuclear development-specific, but with different temporal patterns and absolute expression levels (S3 Fig). Similarly, *O*. *trifallax* encodes twelve Histone H3 variants, six of which are preferentially expressed during macronuclear development (S3 Fig). We confirmed the previously reported expression patterns of the two RPB2 paralogs (S3 Fig) [51].

In order to determine if gene duplication followed by divergence in mRNA expression is widespread in *O*. *trifallax*, we systematically identified paralogous gene sets within the orthogroup dataset in which at least one member was preferentially expressed during macronuclear development. Restricting our analyses to orthogroups with fewer than 20 members in *O*. *trifallax*, there were a total of 2169 orthogroups, of which 233 groups (367 mRNAs) had at least one member that was macronuclear development specific; of these, 183 had at least one member that was not macronuclear development-specific and for the other 50 all members were macronuclear development-specific. Over half of these 233 orthogroups included members associated with the RNA, DNA, chromatin or transcription gene sets (S4 Fig). It is striking how large a fraction of the macronuclear development-specific genes that we identified in these four groups have paralogous members in *O*. *trifallax*; 69 out of 107 RNA group mating-specific mRNAs, 88 out of 150 from the DNA group, 46 out of 61 for the chromatin group and 22 out of 25 from the transcription group. This indicates that over 60% of these macronuclear development specific genes in the four groups arose through gene duplication and specialization. These 233 orthogroups were often phylogenetically conserved (S4 Fig) with over half (131) having a presumptive human homolog. Thus, ~33% of macronuclear development-specific mRNAs encode members of phylogenetically conserved paralogous gene families, many of which encode proteins linked to RNA and DNA biology. Below we highlight two cases in which multiple members of evolutionarily conserved protein complexes underwent duplication and divergence in mRNA expression.

#### RNA polymerase II transcription

**Fig 2:**
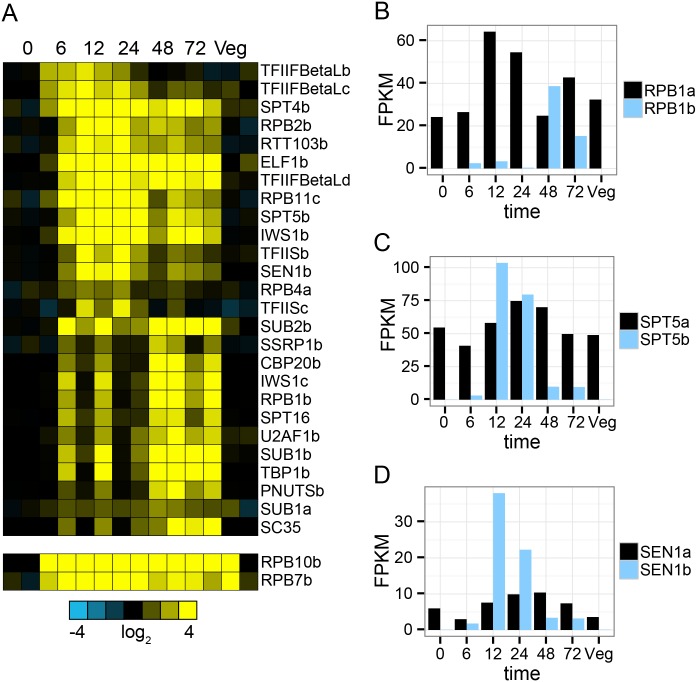
Paralogs of many genes encoding proteins involved in RNA polymerase II transcription are preferentially expressed during macronuclear development. Heatmap representation of expression profiles of mRNAs that were manually curated to RNA polymerase II transcription and which are preferentially expressed during macronuclear development. Genes ending in a-d are part of paralogous gene sets. RPB10b and RPB7b were in module 6, which was removed from macronuclear development-specific gene set due to variable expression in replicates from samples isolated from vegetative cells.Barplot representation of estimated absolute RNA expression levels (FPKM) of the constitutively expressed largest subunit of RNA polymerase II, RPB1a (black), and macronuclear development specific paralog, RPB1b (blue).Same as (b) except for SPT5.Same as (b) except for SEN1.

RNA polymerase II transcribes mRNA precursors and is nearly universally composed of twelve core subunits in eukaryotes, named RPB1-12 in budding yeast [60]. In most species RPBs are singletons and there is a single core RNA polymerase II complex with a suite of other factors that regulate initiation, elongation, termination and processing. However, in *O*. *trifallax* there are two paralogs of RPB1, 2, 4, 7 and 10 and three paralogs of RPB11; in each case one of the paralogs is preferentially expressed during macronuclear development (Fig 2A), and in several cases mRNA levels of the macronuclear development-specific paralog is undetected in vegetative cells (Fig 2B and S5 Fig), but dramatically increases during macronuclear development and rises to levels that rival or exceed that of the constitutively expressed paralog, as shown for RPB1 in Fig 2B. In addition to these core components of RNA polymerase II, a number of factors that assist in initiation, elongation, termination as well as co-transcriptional capping, splicing and polyadenylation have multiple paralogs in *O*. *trifallax* with at least one member preferentially expressed during macronuclear development. These include elongation factors SPT5, SPT4, TFIIS, TFIIF, ELF1 and SSRP1, initiation factors TBP1 and IWS1, and processing factors SUB1, SEN1, RTT103, CBP20, U2AF1, SC35 and SUB2 (Fig 2A). As with the core components, macronuclear development specific accessory proteins are often undetected in vegetative cells and rise to levels on par with the constitutive paralog at one or more stages during macronuclear development (Fig 2C and 2D and S6 Fig). The expression patterns of these mRNAs during macronuclear development fall into two main groups. There is one group whose expression peaks at 12–24 hours (Fig 2A top), but often remains highly expressed throughout macronuclear development, and there is a second group (Fig 2A bottom) that has variable expression at early time points and peaks at 48–72 hours. These results suggest that there may be at least two RNA polymerase II-like complexes with specific roles during macronuclear development in *O*. *trifallax*.

The best characterized example of gene duplication and functional specialization of RNA polymerase II subunits comes from *Arabidopsis* and other flowering plants, which contain two additional multi-subunit RNA polymerases called RNA Polymerase IV and V (reviewed in [61]). These plant-specific RNA polymerase complexes, composed of 12 subunits, have distinct roles in RNA-mediated gene-silencing pathways. Half of the subunits of Pols II, IV, and V are encoded by the same genes, while the remaining Pol IV- or Pol V-specific subunit genes arose through duplication and subfunctionalization of ancestral Pol II subunit genes [62]. Both *O*. *trifallax* and *Arabidopsis* have multiple genes encoding RPB1, RPB2, RPB4 and RPB7, *Arabidopsis* has multiple genes encoding RPB3, RPB5 and RPB9, and *O*. *trifallax* has multiple genes encoding RPB10 and RPB11 (S1 Table). In addition to core RNA polymerase II components, an SPT5 paralog is also a component of the specialized plant RNA polymerase complexes [63].

#### DNA synthesis, recombination and repair

**Fig 3:**
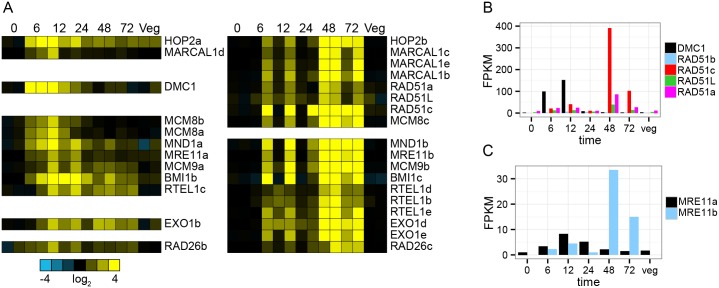
Two sets of paralogs involved in homologous recombination are expressed at different times during macronuclear development. (left) Heatmap representation of expression profiles of paralogs of eleven gene families involved in homologous recombination whose expression levels peak at 6–12 hrs into macronuclear development. (right) Expression levels of other members of these eleven gene families peak at 48 hrs into macronuclear development.Barplot representation of estimated absolute RNA expression levels (FPKM) of RAD51 paralogs.Same as (b) except for MRE11.

We identified 150 mRNAs from our manually curated "DNA" list that are preferentially expressed during macronuclear development and of these, 88 were members of 45 multiple paralog orthogroups in this organism (S3 Fig). Twenty-six of these orthogroups have multiple members preferentially expressed during macronuclear development (S4 Fig). Orthogroups with multiple mating-specific paralogs in *O*. *trifallax* encode a diverse set of evolutionarily conserved factors involved in DNA replication and repair. Expression of DNA genes broadly fell into two groups—one group of ~40 mRNAs peaked early during macronuclear development and another group with ~110 mRNAs peaked at 48–72 hrs (S7 Fig). Many of the genes whose mRNA expression peak at six or 12 hrs encode proteins whose orthologs are core components of homologous recombination pathways associated with meiosis such as MCM8b, MCM9a, HOP2a, MND1a, MRE11a, MARCAL1d, BMI1b, RTEL1c, EXO1b and DMC1 [64, 65]. Strikingly, each of these genes has at least one paralog whose expression peaks at 48–72 hrs into macronuclear development (Fig 3A) and whose estimated maximum absolute expression level is greater (Fig 3B and 3C and S8 Fig), suggesting specialized homologous recombination complex(es) that may be involved in DNA rearrangements. Other orthogroups with macronuclear development-specific members encode DNA synthesis proteins (PCNA1c, POL3b, POL31c), DNA nucleases involved in repair and replication (APEX1c, FAN1b, FEN1b and FEN1c), trans-lesion synthesis polymerase REV1b-e, base excision repair DNA ligase LIG1b, mismatch repair factor MSH6c, DNA topoisomerase TOP2b, condensins (SMC4b, SMC2b/c), and BLM/WRN helicase homolog SGS1b (S7 Fig). Additionally, two gene families appear to have greatly expanded in the *Oxytricha* lineage; the single-stranded DNA binding proteins Replication protein A (RPA) and Poly-(ADP-ribose) polymerase (PARP) (S7 Fig). Five of eight RPA1 paralogs, three of four RPA2 paralogs and seven of sixteen PARPs are preferentially expressed during macronuclear development. RPA is the dominant ssDNA binding protein across eukaryotes with roles in DNA synthesis, repair and recombination [66]. PARPs are multifunctional proteins involved in sensing DNA damage and signaling downstream effectors [67]. Thus, *O*. *trifallax* encodes a rich repertoire of evolutionarily conserved DNA replication and repair factors, whose variable temporal and absolute expression patterns suggest multiple complexes involved in DNA processing during macronuclear development.

### Phylogenetic timing of gene duplication events

To understand the evolutionary timing of gene duplication in RNA polymerase II and homologous recombination factors we performed phylogenetic analyses. For these analyses we retrieved predicted protein sequence files from five additional recently published stichotrich species of varying phylogenetic distance to *O*. *trifallax* (S9 Fig) and the recently sequenced hypotrich *Euplotes octocarinatus* (90% of proteome made available to us), which also has a highly fragmented macronuclear genome and IESs that interrupt MDSs, but no evidence for gene scrambling and with more constrained IES length and pointer sequences [68–71]. We used Orthofinder to retrieve orthogroups with these additional species, manually curated the lists, then performed phylogenetic analysis using maximum likelihood methods with PhyML [72].

**Fig 4:**
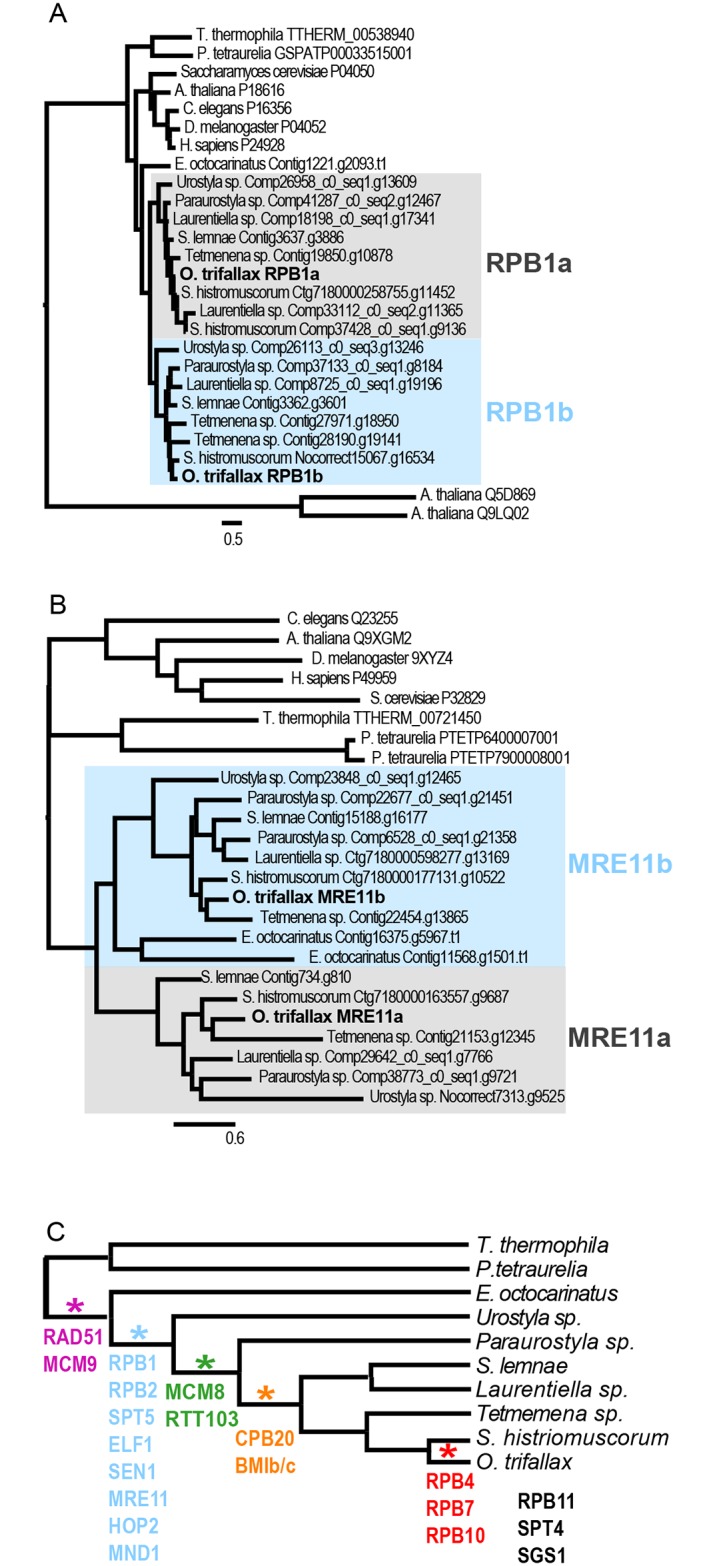
Phylogenetic timing of gene duplication events. Maximum likelihood phylogenetic tree of RPB1. The grey and blue boxes indicate predicted RPB1a and RPB1b paralogs, respectively.Same as (a), except for MRE11. Note that the *E*. *octocarinatus* lineage appears to have had an independent duplication of MRE11, and that *E*. *octocarinatus* MRE11 paralogs group with MRE11b, but with apparent early divergence and long branch length.Estimated timing of gene duplication events overlaid on species phylogenetic tree based on SSU. The color code links the estimated time of duplication with the associated genes.

We generated phylogenetic trees for twelve RNA polymerase II gene families and eight homologous recombination gene families and then gauged the timing of duplication based on bifurcation of the *O*. *trifallax* paralogs. The most common duplication period among these genes appeared to occur after divergence from *E*. *octocarinatus* but prior to divergence of strichotrich species, as judged for five RNA polymerase II genes (RPB1, RPB2, SPT5, ELF1, SEN1) and three homologous recombination genes (MRE11, HOP2, MND1) (Fig 4, S10 and S11 Figs). Note that for SPT5 and MRE11 it is unclear as to whether the duplication event occurred before or after the split with *Euplotes*. RAD51 and MCM9 duplications appear to have occurred prior to divergence of stichotrichs and *Euplotes* (Fig 4C, purple); MCM8 and RTT103 duplications appear to have occurred after divergence of *Urostyla* from other stichotrichs (green); CBC2 and BMIb/c after divergence of *Paraurostyla* from other stichotrichs (orange); RPB4, 7 and 10 appear to have occurred recently, after divergence of *O*. *trifallax* from *S*. *histriomuscorum* (red). We were unable to resolve SPT4, RPB11 and SGS1, and inferred the recent duplication of RPB10 based on 100% amino acid identity between paralogs. We conclude all gene duplication events occurred after the divergence of spirotrich and oligohymenophorea lineages. Within the spirotrich lineage duplications commonly preceded the divergence of strichotrich species, but is an ongoing process.

### Dozens of evolutionarily conserved germline factors are core components of macronuclear development programs in ciliates

While separated by over one billion years of evolution, the distantly related ciliates *Paramecium* and *Tetrahymena* also have macronuclear developmental programs that involve site-specific DNA elimination and transection of chromosomes and it is that likely some of the machinery required for macronuclear development existed in their common ancestor with *O*. *trifallax*. We reasoned that mRNAs preferentially expressed during macronuclear development in *O*. *trifallax* and *P*. *tetraurelia* or *T*. *thermophila* may encode a core group of proteins involved in the ancestral developmental program whose functional themes highlight fundamental features of the program. It is also a distinct possibility that many of these proteins are broadly involved in germline development and germline-soma stratification. For instance, PIWI related pathways are not only key players in macronuclear development in all three species, but are now well-recognized to have important roles in germline maintenance and propagation across eukaryotes [73].

**Fig 5:**
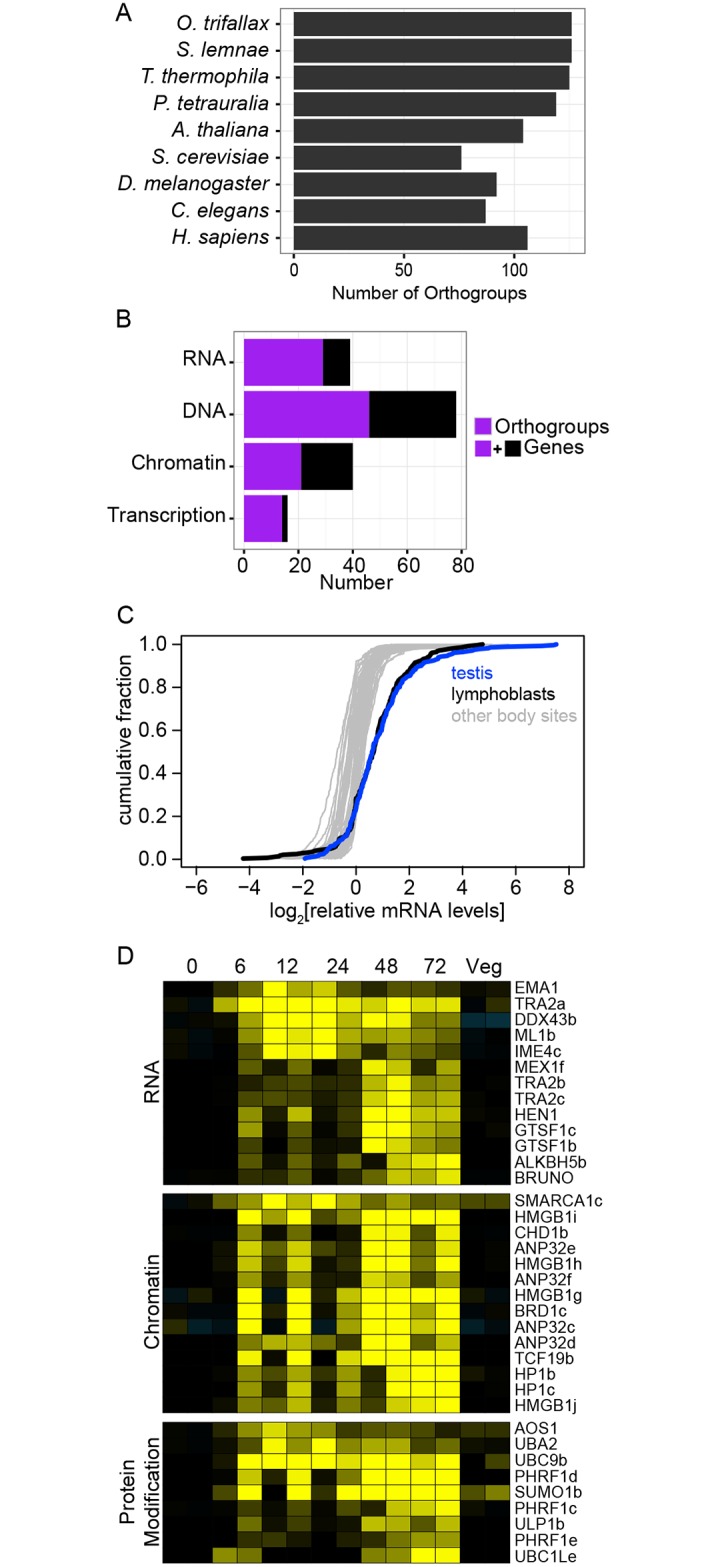
Dozens of evolutionarily conserved germline factors are core components of macronuclear development programs in ciliates. Barplot representation of the number of orthogroups in which at least one member was preferentially expressed during macronuclear development in *O*. *trifallax*, had a representative in *S*. *lemnae*, and in which at least one member was preferentially expressed during macronuclear development in *T*. *thermophila* or *P*. *tetraurelia*; for other species analyzed the number of those orthogroups with at least one member is plotted.Barplot representation of the number of orthogroups (purple) and genes (purple + black) from the orthogroups identified in (a) that were associated with four manually curated gene lists associated with RNA and DNA biology.Cumulative fraction plots of relative mRNA expression of human orthogroup members from (a) across 53 body sites. Testis (blue) and *ex vivo* cultured lymphocytes (black) were shifted to the right relative to other sites.Heatmap representation of relative expression of mRNAs identified in (a) annotated to “RNA” (top), “chromatin” (middle) and “protein modification” (bottom).

We identified mRNAs that are differentially expressed during macronuclear development in *P*. *tetraurelia* and *T*. *thermophila* using published microarray expression profiles [74, 75] and intersected these gene sets with orthogroups in which at least one member was preferentially expressed during macronuclear development in *O*. *trifallax*. We identified 126 orthogroups (110 orthogroups intersected with *T*. *thermophila*, 72 with *P*. *tetraurelia* and 56 with both), including several gene groups with seminal roles in macronuclear development in *Paramecium* and *Tetrahymena*, such as RNAi components PIWI, DICER and RDRP, and Histone H3 variants (S5 Dataset). These 126 orthogroups were generally not restricted to ciliates, with 76 and 106 also having at least one homolog in budding yeast and humans, respectively (Fig 5A). The genes in these 126 orthogroups are functionally diverse, but a disproportionate number encode proteins implicated in themes highlighted already, including RNA (29), DNA (46), chromatin (21) and transcription (14) (Fig 5B). These include homologs of many of the aforementioned RNA polymerase II and DNA synthesis and repair proteins (SDataset 3). The most prevalent protein domains encoded by representative genes in these orthogroups were AAA (15), C3HC4 type Zinc finger (10), PHD-Finger (9), RRM (6) and DEAD/H box helicase (5).

Macronuclear development programs in ciliates share many features of germline cell specification and differentiation in multicellular organisms. That such a large fraction of the genes identified above are broadly conserved outside the ciliate lineage raises the question as to whether their homologs in multicellular organisms tend to be preferentially expressed in germline cells and involved in germline functions. To test this hypothesis, we retrieved the Genotype-Tissue Expression (GTEX) RNA sequencing dataset [76] which profiled multiple samples from 53 human body sites and plotted the relative mRNA expression levels of the 283 (106 groups) human homologs as a function of body site (Fig 5C). The relative expression profile for each body site is represented by its cumulative distribution; while 51 of the samples were largely overlaid, testis (blue) and EBV-transformed lymphocytes (black) were significantly shifted to the right due to higher relative expression of these mRNAs as a whole. mRNAs preferentially expressed in lymphocytes were mostly linked to DNA synthesis and repair and their expression may reflect higher proliferation rate of these cells, which were immortalized and cultured *ex vivo*, compared to other body sites (S12 Fig). Most of these mRNAs were also preferentially expressed in testis. Additionally, there was a set of ~30 mRNAs whose expression was >2 fold higher in testis relative to all other body sites (S12 Fig). These mRNAs include PIWIL2 and the PIWI pathway factors 2’O methyltransferase HEN1 and gametocyte-specific factor 1 (GTFS1), which was proposed to be a central component of nuclear PIWI effector complexes in *D*. *melanogastor* [77]. *O*. *trifallax* HEN1 mRNA expression peaks at 48–72 hrs into macronuclear development (Fig 5D); HEN1 in other species, including *T*. *thermophila*, adds 2’O methyl groups to 3’ terminal nucleotide of PIWI associated small RNAs [78, 79]; however, published studies on *O*. *trifallax* macronuclear development-specific 27mer PIWI associated 27macRNAs showed no evidence for 2’O methylation [38, 39].Testis-specific genes ELF1 and TFIIS homolog TCEA2 are additionally proposed to be involved in piRNA biogenesis in *D*. *melanogaster* [80]. This set also includes ALKBH5 RNA demethylase, which is linked to fertility in mouse [81], two poorly characterized but well-conserved RNA helicases DDX43 and DDX53, a nuclear cap binding protein paralog NCBP2L, an SPT5 homolog and JADE3 histone acetyl transferase among others. Many of these genes are poorly characterized, but given their broad conservation and testis-specific expression pattern in humans, warrant further investigation.

Manual curation of the dataset revealed an abundance of additional factors linked to germline related processes. We highlight genes encoding proteins involved in RNA metabolism, chromatin modification and SUMOylation in S1 Text (Fig 5D).

### Identification of recently acquired DNA and RNA-binding proteins with potential roles in macronuclear development

**Fig 6:**
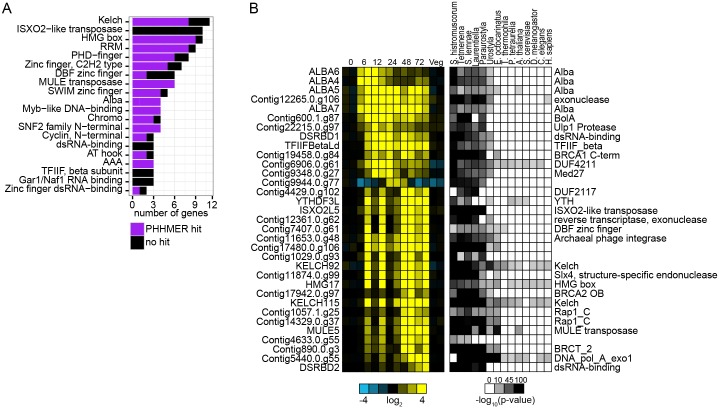
Recently acquired genes encoding proteins with potential roles in macronuclear development. Barplot showing protein domains commonly found in mRNAs preferentially expressed during macronuclear development but which do not have an orthogroup member outside of *S*. *lemnae*. Purple shows the number that had a match to at least one gene in any of the other seven stichotrich species and black shows the number that did not have a significant match.(left) Heatmap representation of relative mRNA expression of 33 selected mRNAs from the analyses described in (a). (right) Heatmap representation of Orthofinder BLAST p-values for genes on the left with five additional stichotrich species and the hypotrich *E*. *octocarinatus*. To the right of the list are protein domains associated with said genes identified with HMMER.

While we have largely focused on genes whose protein products are broadly conserved across eukaryotic lineages, it is likely that the advent of the dramatic genome reorganization and resolution in the *Oxytricha* lineage also involved acquisition and evolution of novel genes. We identified such genes as those preferentially expressed during macronuclear development and with an orthogroup member in *S*. *lemnae*, but in none of the other species. 433 genes (385 orthogroups) met these criteria. We gauged sequence identity of these 433 proteins against the other seven model organisms’ proteomes using PHMMER [82]. One hundred and thirty-three proteins bore similarity to at least one protein in one of the other species considered (e-value ≤ 0.01); in most cases the similarity was limited to a single domain in the protein. Thus, most of these genes appear to encode proteins with limited phylogenetic distribution. Common protein domains found in these 433 proteins included Kelch (11), HMG-box (10), RRM (9), ISXO2-like transposase (10), MULE transposase (6), Alba (4), and a number of other DNA- and RNA-binding domains (Fig 6A). We selected a subset of 33 of these genes based on their mRNA expression levels and predicted domains and looked more closely at their potential functions as well as their conservation across recently sequenced stichotrichs and *E*. *octocarinatus* (Fig 6B). Many of these proteins with restricted phylogenetic conservation whose expression is limited to macronuclear development appear to function in RNA and DNA processes, implying potential roles in DNA rearrangements (Details are described in S2 Text).

### Identification of regulatory elements that specify macronuclear development gene expression programs

The macronuclear development-specific mRNA expression profiles imply the existence of associated *cis*-acting regulatory elements. In order to identify such elements, we retrieved regions immediately upstream and downstream of annotated coding sequences, and searched for linear sequence motifs significantly enriched in these elements for the genes in modules 1–5 using HOMER [83]. At the suggested p-value threshold of 1e-11 we identified ten upstream motifs (module 1–5: 2,3,1,3,1 respectively) and four downstream motifs (modules 1–5: 0,3,0,1,0 respectively) (S13 Fig).

The most significant motif is enriched upstream of coding sequences in module 4. It is a 12mer in which positions 6–10 are palindromic to 1–5, as is commonly observed among transcription factors that bind as dimers. It is found in 55 of 423 module 1 promoters (13%) vs 1.4% of background promoters. The motif tends to occur ~100 nucleotides upstream of the annotated translation start site (median = 102), a significantly different distribution than seen in background sequences (median = 150, two-sided K-S test, p = 0.0006) (S13 Fig). One way to infer functionality of a potential regulatory element is to see if it is conserved in orthologous sequences in closely related species. Among the available stichotrich genomes, *S*. *histriomuscorum* is most closely related to *O*. *trifallax*. We find that among 187 *S*. *histriomuscorum* orthologs in module 4 the motif is found in 14, significantly more than expected by chance (hypergeometric distribution, p = 1e-6). We identified a similar motif as most significant in these 187 *Sterkiella* sequences using HOMER (p = 1e-11) (S13 Fig). These results argue for the functionality of this motif as a regulatory element. In S3 Text we further characterize two of the other putative regulatory elements. While preliminary, this work provides the first examples of potential cis-acting regulatory elements in *O*. *trifallax*.

### The micronuclear genome contributes hundreds of genes to the macronuclear development expression program

The micronuclear genome is generally considered quiescent and is dispensable for vegetative growth; however, a recent study demonstrated that hundreds of functional mRNAs are transcribed from DNA sequences that are present in the micronuclear genome but which are not processed to become part of the macronuclear genome. These micronuclear-limited genes are interesting in that they were only observed to be expressed during macronuclear development, not during vegetative growth [24]. We retrieved the coding sequences for these 810 mRNAs and quantified their expression levels in our dataset using a modified workflow [84]. We found that 259 of these mRNAs were reliably detected in at least one sample (at a similar expression threshold to what was employed above; three transcripts per million (TPM) in at least one sample). In concert with the published work we find that expression of these mRNAs is categorically limited to macronuclear development with expression of most of these 259 mRNAs peaking at 48 or 72 hrs with variable expression at the earlier time points (S14 Fig). An abundance of these 259 mRNAs encode proteins linked to DNA and RNA functions, mirroring the themes seen in our main dataset (S12 Fig). Common domains include 13 with MT-70 m6A methyltransferase, five histones, four HMG-box, four PHD and three tudor-knot (S14 Fig). These results support the published work that the micronuclear genome encodes hundreds of proteins that contribute to macronuclear development.

## Discussion

Identifying the mechanisms by which the single-celled ciliate *O*. *trifallax* evolved two remarkable genomes segregated for germline and somatic functions and transforms the scrambled, nonfunctional germline genome into the highly streamlined functional somatic genome after sexual reproduction would provide insights into the principles underlying evolutionary innovation. The mRNA expression profiles of macronuclear development in *O*. *trifallax* presented herein implicate a staggering array of DNA and RNA-interacting proteins in this process. From our work, we hypothesize that the specialized function of genes involved in macronuclear development is acquired via several mechanisms: (i) a core set of factors involved in both macronuclear development across ciliates and germline-soma stratification in multicellular organisms (Fig 4), (ii) expansion of protein families with domains involved in DNA and RNA metabolism (Fig 1), (iii) specialization of multimember DNA and RNA-binding protein machines via gene duplication (Figs 2 and 3) and (iv) recent acquisition of diverse DNA and RNA remodeling factors, such as through transposon domestication (Fig 5) [26, 85].

The identification of telomerase, specialized histone H3 and histone acetyl transferase in *T*. *thermophila* as well as seminal studies on PIWI and HP1 proteins, and the subsequent characterization of their importance in germline development and germline-soma stratification provided direct molecular connections between nuclear duality in ciliates and germline-soma specialization in multicellular organisms. We found that the mRNAs encoding ~120 protein families that are preferentially expressed during macronuclear development in *O*. *trifallax* and also in the distantly related ciliates *T*. *thermophila* and/or *P*. *tetraurelia* are generally not ciliate specific, are preferentially expressed in germline tissue in humans (testis) and are commonly linked to germline functions (Fig 5). These results suggest fundamental commonalities in the protein machinery and the underlying mechanisms between nuclear duality in ciliates and germline-soma stratification in multicellular organisms. These ~120 protein families may form a core set of factors involved in sexual development and germline specialization. Indeed nuclear dimorphism in ciliates may have been a test case of germline-soma stratification, utilizing many of the same components used in metazoans and plants [86]. The specific functions of these proteins in *O*. *trifallax* are unknown, but promise to provide insights into the developmental program *per se* and into how the specific roles of these proteins change over time. For instance, in *T*. *thermophila* and *P*. *tetraurelia* PIWI-associated RNAs derive from the micronuclear genome and are believed to mark IESs for deletion whereas in *O*. *trifallax* they originate from the macronuclear genome and mark MDSs for retention [16, 38, 39, 87]. Pdd1 in *T*. *thermophila* assists PIWI in marking IESs for deletion, while the HP1b/c expression pattern in *O*. *trifallax* suggests they play a different role, such as marking the bulk of germline DNA for elimination. The expression patterns of many of these factors during macronuclear development in *O*. *trifallax* differ in timing and level of induction from from their *T*. *thermophila* and *P*. *tetraurelia* homologs, suggesting functional divergence. As germline cells are generally rare and difficult to isolate and study, further efforts in ciliates could provide broad insights into the logic of these programs and functions of specific protein families, many of which are still poorly characterized.

Gene duplication was first hypothesized to be an important mechanism of evolutionary innovation decades ago [88], and our appreciation of its pervasive role in evolution has greatly expanded in the genomic age [59]. We identified a slew of gene duplication events that may have facilitated the evolution of *O*. *trifallax’s* macronuclear development program (Figs 2–4). One flavor of gene duplication we described is the expansion of specific protein families, often linked to DNA and RNA functions. Members of these protein families, including the previously characterized PIWI and Histone H3, as well as Zinc fingers, HMG box, PHD-finger, RRM, DEAD/H helicase, PARP, RPA, Alba, Kelch, ISXO2 and MULE transposases, tend to be preferentially expressed during macronuclear development, often at different times and absolute levels (Fig 1). Expansion of these protein families may constitute subfunctionalization whereby duplication events enabled members to take on additional or more specialized functions. For instance, the RPA heterotrimer binds single-stranded DNA intermediates in many programs including DNA replication, homologous recombination and DNA repair [66]. *O*. *trifallax* has the richest repertoire of RPA1 and RPA2 genes to date (S7 Fig), suggesting specialized RPA trimers that recognize and signal different forms of single-stranded DNA intermediates; for instance, from RNA-DNA hybrids between PIWI:27mer RNA complexes or long RNA templates and MDSs during MDS recombination and from intermediates in gene excision and the multiple phases of DNA replication that occur during macronuclear development (S1 Fig). HMG box proteins often bind and bend specific noncanonical DNA structures and may be required to sculpt DNA topology during DNA rearrangements [89, 90]. The expansion of diverse nucleic acid-binding protein families, often with modular recognition domains, underscores the scope of RNA-mediated DNA processing events during macronuclear development. We speculate that stichotrichs’ propensity for shuffling pieces of DNA around in the micronucleus may facilitate gene duplication.

The second flavor of gene duplication we described involves sets of genes encoding proteins that function together as multimeric complexes (Figs 2 and 3). Coordinate divergence in mRNA expression patterns of duplicated gene pairs of members of these complexes suggests functional specialization. Duplication of many of these factors preceded the divergence of stichotrich species and may have played fundamental roles in the evolution of the macronuclear development program (Fig 4, S10 and S11 Figs). The paralogous RNA polymerase II factors that are expressed specifically during macronuclear development cover the gamut of transcription-related functions and many are among the most abundant mRNAs at early time points (Fig 2, S3 and S4 Figs). Analogous to RNA polymerase IV and V complexes in flowering plants, we suggest there are at least two macronuclear development specific RNA polymerase II-derived complexes, one of which is responsible for the *en masse* bidirectional transcription of the macronuclear genome during the early stages of macronuclear development to produce long dsRNAs and precursors and/or templates for PIWI 27macRNA production (Fig 2) [37–39]. Landweber and colleagues proposed that the macronuclear development-specific paralog RPB2 does not function in bidirectional transcription of the macronuclear genome, but rather acquired dsRNA binding capability and plays a role in trafficking template RNA between nuclei [51]. While our results do not counter this hypothesis *per se*, we suggest a direct role in bidirectional transcription is more parsimonious. Based on mRNA expression patterns, the second macronuclear development-specific RNA polymerase II-derived complex, which includes a paralog of the largest subunit RPB1, functions during the latter stages of macronuclear development (Fig 2). We hypothesize this complex transcribes either the micronuclear genome-derived mRNAs (S14 Fig) [24] or an unidentified class of noncoding RNAs.

The second example of multicomponent complexes whose subunits are duplicated and show macronuclear development-specific expression involves genes encoding proteins involved in DNA recombination, repair and synthesis (Fig 3 and S5 Fig). There are two paralogous sets of factors involved in homologous recombination, one whose expression peaks early during macronuclear development and is likely involved in crossover events during meiosis of the parental micronucleus, and a second set which peaks later, coinciding with the bulk of DNA rearrangements. These events are presumed to involve RNA-DNA pairing, or R-loops, which are not canonically associated with homologous recombination and DNA repair machinery. However, recent studies in diverse systems point to functional connections between noncoding RNAs, R-loops, and homologous recombination and repair machinery [91, 92]; for instance, R-loop formation initiates the pathway of V(D)J recombination [93], RNA is able to serve as a template for repair of damaged DNA in yeast [94], small RNA induced quelling in *Neurospora* requires homologous recombination proteins [95] and Rad51 promotes R-loop formation in *trans* [96]. We hypothesize that the homologous recombination and repair proteins whose expression coincides with DNA rearrangements utilize RNA templates and participate in MDS recombination, repair and gene excision.

What are the roles of the dozens of recently acquired genes whose expression levels peak during DNA rearrangements? Many have domains that suggest roles in DNA manipulations including the previously identified domesticated MULE and ISXO2 transposases, as well as proteins with similarity to phage integrase, SLX4 recombinase, reverse transcriptase and DNA exonucleases, while others are completely foreign (Fig 6). Given the timing, level of expression and evolutionary conservation among strichotrichs and spirotrichs, respectively, it is feasible that the ISXO2 and MULE transposases participate in excision of genes from chromosomes. While their functions and enzymatic activities are now obscure, one of more of these proteins could eventually be utilized for future generations of genome editing, for instance by improving the rate of homologous recombination of CRISP-CAS9 systems or via directed RNA-guided DNA repair.

Ciliates have generally lived on the fringes of molecular and genomic research due their abnormal genetic properties. The work presented herein reinforces the vision of Prescott and others that understanding the genetic apparatus of *Oxytricha*, and ciliates in general, will inform our appreciation of genome organization, differentiation, inheritance and evolution and identify new mechanisms to manipulate genomes [97].

## Materials and methods

### Vegetative growth of *Oxytricha trifalla*x

*O*. *trifallax* strains ALXC2 and ALXC9 [38] were grown vegetatively in Pyrex dishes in inorganic salts media [98] using the food source *Chlorogonium elongatum* (UTEX collection strain B203). Typical daily feedings consisted of 10 mL of washed algae per 300 mL Pyrex dish of *O*. *trifallax*, depending on culture density.

### Mating of Oxytricha trifallax

Mating competent strains ALXC2 and ALXC9 were grown vegetatively to high density, fed lightly the day before a mating and allowed to completely exhaust their food supply. Cells were then cotton filtered to remove any residual algae and were concentrated on 10 μM Nitex membranes into Pringhsheim salts media [39]. The concentration of cells in each individual mating strain was determined and cells were then mixed at equal numbers to a total concentration of 1,500 cells per mL in Pyrex dishes. These mating cells were then fed 1 mL of unwashed *Klebsiella pneumonia* stationary phase culture as a food source. Aggregates of 10–30 cells were observed by 2 hours after mixing mating types, with the first mating pairs visible by 4 hours post mixing. Typical mating efficiency when mixing ALXC2 and ALXC9 strains is ~70%, with visible developing macronuclei present by 48 hours.

### Total RNA isolation

Mating cells were filtered through cotton to remove cellular debris, concentrated onto 10 μM Nitex membranes and transferred into microcentrifuge tubes. Cells were gently pelleted by centrifugation for 2 minutes at 500 x g in a microcentrifuge and supernatants were removed, leaving ~50 μL of pelleted cells. 200 μL of mirVana Lysis/Binding Buffer from the mirVana miRNA Isolation Kit (ThermoFisher) was added to each tube, mixed, and total RNA was purified using the kit’s protocol for total RNA purification. Total RNA yield from a single 300 mL Pyrex dish of cells (~450,000 cells) was typically between 100 and 300 μg.

### Illumina sequencing

Sequencing libraries were prepared using the TruSeq RNA Sample Preparation v2 Kit (Illumina) following the manufacturer’s LT protocol. Library preparation started with 3 μg of total RNA from each sample. Individual libraries were analyzed on a Bioanalyzer (Agilent), then pooled for sequencing. Pooled libraries were sequenced on an Illumina HiSeq 2000 at the UCSC Genome Technology Center generating 100bp paired-end reads. FASTQ files of raw sequencing reads from 14 sequencing libraries are available through Gene Expression Omnibus (accession GSE86081).

### Alignment and post-alignment processing

Raw sequencing results in the form of FASTQ files were used as input for alignment of the sequencing data to the *O*. *trifallax* macronuclear genome reference RNA database [26]. The reference RNA GTF and macronuclear genome FASTA files were retrieved from the *Oxytricha* genome website (oxy.ciliate.org). Alignments were performed with Tophat2 using default options, which produced BAM alignment files [55]. Tophat BAM files were used directly as input into Cuffdiff2 to generate normalized FPKM values [56].

### Sequencing data analyses

Gene-centric normalized FPKM values were filtered as follows: mRNAs in which FPKM was not ≥ 3 in at least one sample were removed, remaining mRNAs were log_2_ (FPKM +1) transformed, and each mRNA was then normalized by subtracting the mean log_2_ (FPKM+1) value of 0 hour time points. mRNAs preferentially expressed during macronuclear development were defined as those whose average relative mRNA expression in one of the developmental time points was at least 3-fold greater than average 0 hour and vegetative cells. Normalized relative expression of these 1162 mRNAs was input for WGCNA (power = 22, networkType = signed, minModuleSize = 50, reassignThreshold = 0, mergeCutHeight = 0.25) [58], which produced six modules. One module was removed because mRNAs were highly expressed in one of the two samples from vegetative cells. Modules were ordered by temporal expression and mRNA similarity within modules was determined with Cluster 3.0 using average-linkage centered Pearson correlation and results were visualized with Java Treeview [99].

### Quantification of micronuclear genome encoded mRNAs

Coding sequences for 810 micronuclear-derived mRNAs plus annotated macronuclear RNAs were combined and expression levels (TPM) were determined with Kallisto under default settings [84]. TPM values were filtered as follows: mRNAs in which TPM was not > 3 in at least one sample were removed, remaining mRNAs were log2 (FPKM +1) transformed, and each mRNA was then normalized by subtracting the mean log2 (FPKM+1) value of 0 hour time points.

### Protein domain and gene ontology analyses

Protein domains and GO annotations were retrieved from the *Oxytricha* genome website. The p-values of enrichment of protein domains and GO terms in specific gene sets were determined using the hypergeometric density distribution function and corrected for multiple hypothesis testing using the Benjamini-Hochberg method [100]. Additional domain and sequence identity searches were performed with HMMER v3.1 [82, 101].

### Identification of orthogroups

Orthogroups were defined using Orthofinder with default settings [57]. *O*. *trifallax*, *S*. *lemnae*, *T*. *thermophila* and *P*. *tetraurelia* protein FASTA files were obtained from their respective genome websites. FASTA files for *S*. *cerevisiae*, *A*. *thaliana*, *D*. *melanogaster*, *C*. *elegans* and human were obtained from the InParanoid website (inparanoid.sbc.su.se). Gene annotations were obtained from genome site SQL tables for *T*. *thermophila* (www.ciliate.org), from the genome sites for *P*. *tetraurelia* (www.paramecium.cgm.cnrs-gif.fr) and *S*. *cerevisiae* (http://www.yeastgenome.org) and from Uniprot for *A*. *thaliana*, *D*. *melanogaster*, *C*. *elegans* and human (www.uniprot.org).

### Curated gene lists

Genes associated with RNA functions (“RNA”), DNA synthesis and repair (“DNA”), RNA polymerase II transcription (“transcription”) and chromatin (“chromatin”) were identified as follows:

#### RNA

We retrieved genes annotated as RNA-binding proteins in humans and common RNA-binding domains [102] and genes associated with RNA metabolism in *S*. *cerevisiae* [103]. A compendium of *O*. *trifallax* genes linked to RNA biology was generated by first identifying orthogroups in which members of the orthogroup were annotated as RNA-binding proteins in human or *S*. *cerevisiae*. To this set we added genes with canonical RNA-binding domains or whose GO or protein domain annotation included “RNA”. We added a handful of genes based on sequence similarity to genes linked to RNA functions. This set was manually filtered to remove genes that did not appear to directly be associated with RNA functions.

#### DNA

We retrieved genes associated with “DNA repair” and “DNA replication” in humans from Reactome database (www.reactome.org) and retrieved genes associated with “DNA repair”, “DNA replication” and “DNA recombination” in *S*. *cerevisiae* from Saccharomyces Genome Database (http://www.yeastgenome.org). A compendium of *O*. *trifallax* genes was generated by first identifying orthogroups in which members of the orthogroup were in the lists above. To this set we added genes whose GO or protein domain annotation included “DNA”. This set was manually filtered to remove genes that did not appear to directly be associated with DNA replication, repair or recombination.

#### Transcription

We retrieved genes associated with “RNA polymerase II Transcription” in humans from the Reactome database. We retrieved genes associated with “core RNA polymerase II recruiting transcription factor activity”, “RNA polymerase II core promoter sequence-specific DNA binding”, “RNA polymerase II core promoter sequence-specific DNA binding transcription factor activity involved in preinitiation complex assembly”, “DNA-directed RNA polymerase activity” in S. *cerevisiae* from Saccharomyces Genome Database. To this set we added genes whose GO or protein domain annotation included “transcription”. This set was manually filtered to remove genes that did not appear to directly be associated with RNA polymerase II transcription.

#### Chromatin

We retrieved genes associated with “chromatin organization” in humans from the Reactome database. We retrieved genes associated with “chromatin modification” and “chromatin remodeling” in *S*. *cerevisiae* from Saccharomyces Genome Database. Histone genes in *S*. *lemnae* were retrieved from the macronuclear genome sequence manuscript [7] and via BLAST searches against the *O*. *trifallax* genome. We added genes whose GO or protein domain annotation included “chromatin” and genes with bromo or chromo domains. This compiled gene set was then manually filtered.

### Gene naming conventions

Gene naming largely focused on orthogroups whose members were annotated to one of four groups described above and/or for which at least one member was preferentially expressed during macronuclear development. Emphasis was placed on naming genes with orthogroup members in one or more of the non-stichotrich species. Generally, *O*. *trifallax* genes within an orthogroup were ordered by absolute expression level in vegetative cells (based on average FPKM) and names were guided by names associated with orthogroup members with emphasis on human and yeast members. The member of the orthogroup with highest absolute expression in vegetative cells was assigned the name GENEa, second highest GENEb and so on. Gene names are available on *Oxytricha* Genome Database.

### Identification of orthogroups including additional spirotrich species and phylogenetic tree generation

Orthogroups were defined using Orthofinder. Predicted non-programmed ribosomal frameshifting protein sequences for *E*. *octocarinatus* were provided by Professor Aihua Liang (Shanxi University, China) [69]. Predicted protein sequences for *Urostyla sp*., *Paraurostyla sp*., *Laurentiella sp*., *Tetmemena sp*., and *S*. *histriomuscorum* were provided by Dr. Xiao Chen (Princeton University) [68].

Multiple sequence alignments were performed using Clustal-Omega 1.1.0 Multiple Alignment on the Mobyle@Pasteur portal (http://mobyle.pasteur.fr/cgi-bin/portal.py#forms::clustalO-multialign) [104]. These Clustal-Omega multiple sequence alignments were then run through PhyML Version 3.0 with a single substitution rate category and the Le and Gascuel (LG) substitution model [105], or in the case of the small-subunit ribosomal RNA tree the HKY85 nucleotide substitution model [106], optimized for tree topology, branch length and rate parameters on the Mobyle@Pasteur portal (http://mobyle.pasteur.fr/cgi-bin/portal.py#forms::phyml) to create maximum likelihood phylogenetic trees [107]. One hundred bootstrap sets were used for all of the multiple sequence alignments analyzed. The maximum likelihood Newick tree files produced using PhyML were then uploaded into the tree viewer FigTree 1.4.2 (http://tree.bio.ed.ac.uk/software/figtree, last accessed August 3, 2016). The tree images incorporate branch lengths, but bootstrap values were not included in the tree images for the sake of visualization.

### *Tetrahymena* and *Paramecium* microarray data

*T*. *thermophila* microarray data were obtained from Gene Expression Omnibus (GSE11300) [74]. Normalized signal intensities for each experiment were log_2_ transformed and mRNAs with values of less than seven in all experiments were removed. Data were normalized relative to mean values from the starvation time points (“S”). mRNAs preferentially expressed during macronuclear development were defined as those whose relative abundance was at least 3-fold greater in any of the macronuclear development time points, on average, versus the average of starvation time points and average of vegetative samples.

*P*. *tetraurelia* microarray data were obtained from Gene Expression Omnibus (GPL7221) [75]. Multiple probes corresponding to a particular gene were collapsed by their geometric mean, log_2_ transformed and mRNAs with values of less than nine in all experiments were removed. Data were normalized relative to mean values from the vegetative samples. mRNAs preferentially expressed during macronuclear development were defined as those whose relative abundance was at least 3-fold greater in any of the macronuclear development time points, on average, versus average of vegetative samples.

### Human GTEX data analyses

Gene centric reads per kilobase million (RPKM) data (GTEx_Analysis_2014-01-17_RNA-seq_RNA-SeQCv1.1.8_gene_rpkm.gct) were obtained from the GTEX website (www.gtexportal.org/). RPKM data were log_2_+1 transformed, mRNAs were mean centered, and samples from each body site were collapsed to their mean.

### Motif finding

Linear sequence motifs overrepresented upstream and downstream of coding sequences of mRNAs in modules 1–5 were identified with HOMER v4.8 [83]. FASTA files containing up to 500 bases upstream (or downstream) of coding sequences were generated using BEDTools [108], sequences less than 80 bases in length were removed and sequences were filtered to remove overlapping coding sequences and telomere sequences. Sequences from mRNAs with reliable signal (FPKM ≥ 3 in at least one experiment), but not defined as preferentially expressed during macronuclear development were used as background.

*S*. *histriomuscorum* orthologs were defined using InParanoid v 4.1 [109]. The macronuclear genome sequence FASTA file and gff annotation file were provided by Dr. Xiao Chen and upstream and downstream sequences were extracted as described above for *O*. *trifallax*.

## Supporting information

S1 Fig *O*. *trifallax* sexual life cycle. **1.** Two vegetative *O*. *trifallax* cells of different mating types (represented by the difference in nuclei colors). **2.** Under starvation conditions the two cells fuse and begin to conjugate. 3. A parental micronucleus in each cell undergoes meiosis. **4–6.** Three of the newly formed haploid micronuclei will break down while the remaining one will undergo mitosis. One mitosis-derived haploid micronucleus is exchanged between the mating cells. **7.** The newly acquired micronucleus fuses with the remaining maternal micronucleus to become diploid. **8.** The newly formed diploid micronucleus undergoes mitosis. **9.** One newly formed micronucleus develops into a new macronucleus while the maternal macronucleus is broken down and degraded. **10.** Two genetically identical exconjugant *O*. *trifallax* cells. Inside the circle is a graph showing the general timing of macronuclear development events in hours and the corresponding DNA content of the developing macronucleus. Outside of the circle are the alternate microculear and macronuclear versions of two hypothetical genes, one scrambled and one nonscrambled (MDSs in orange, IESs and nongenic DNA in blue and telomeres in black) [20, 29, 31]
(TIF)

S2 Fig Distribution of members of curated gene lists among modules 1–5. Barplot representation of the percentage of macronuclear development-specific members of each of the four gene lists in modules 1–5.
(TIF)

S3 Fig Expression profiles of PIWI, Histone H3 and RPB2 paralogs during macronuclear development. Heatmap representation of mRNA expression profiles of PIWI paralogs preferentially expressed during macronuclear development.Barplot representation of estimated absolute RNA expression levels (FPKM) of PIWI paralogs.Same as (a) except for Histone H3.Same as (b) except for Histone H3.Same as (b) except for RPB2.
(TIF)

S4 Fig An abundance of mRNAs encoding paralogous proteins involved in RNA and DNA metabolism are preferentially expressed during macronuclear development. Barplot representation of the number of orthogroups (purple) and genes (purple + black) from the orthogroups identified in (b) that were associated with four manually curated gene lists associated with RNA and DNA biologyBarplot representation showing orthogroups containing at least two members in *Oyxtricha* in which at least one member is preferentially expressed during macronuclear development; for the other species analyzed, the barplot shows the number of those orthogroups with at least one member in said species.
(TIF)

S5 Fig Estimated expression levels of RNA polymerase II paralogs during macronuclear development. Barplot representation of estimated absolute RNA expression levels (FPKM) of RPB1 paralogs during macronuclear development.Same as (a) except for RPB2.Same as (a) except for RPB4.Same as (a) except for RPB7.Same as (a) except for RPB10.Same as (a) except for RPB11.
(TIF)

S6 Fig Estimated expression levels of RNA polymerase II accessory factors during macronuclear development. Barplot representation of estimated absolute RNA expression levels (FPKM) of CBP20 paralogs expressed during macronuclear development.Same as (a) except for ELF1.Same as (a) except for IWS1.Same as (a) except for PNUTS.Same as (a) except for RTT103.Same as (a) except for SEN1.Same as (a) except for SPT4.Same as (a) except for SPT5.Same as (a) except for SSRP1.Same as (a) except for SUB1.Same as (a) except for SUB2.Same as (a) except for TBP1.Same as (a) except for TFIIFBeta.Same as (a) except for TFIIS.Same as (a) except for U2AF1.
(TIF)

S7 Fig Paralogs of many genes involved in DNA synthesis, repair and recombination are preferentially expressed during macronuclear development. Barplot showing DNA synthesis, repair and recombination genes with multiple members in *O*. *trifallax* in which at least one member is preferentially expressed during macronuclear development. Blue shows the number that are preferentially expressed during macronuclear development, black shows the number that are not preferentially expressed during macronuclear development.Heatmap representation of mRNA expression profiles of DNA synthesis, repair and recombination genes preferentially expressed during macronuclear development.Heatmap representation of mRNA expression profiles of RPA and PARP gene family members preferentially expressed during macronuclear development.
(TIF)

S8 Fig Estimated expression levels of DNA synthesis, repair and recombination factors during macronuclear development. Barplot representation of estimated absolute RNA expression levels (FPKM) of BMI paralogs during macronuclear development.Same as (a) except for EXO1.Same as (a) except for HOP2.Same as (a) except for MARCAL1.Same as (a) except for MCM8.Same as (a) except for MCM9.Same as (a) except for MDN1.Same as (a) except for MRE11.Same as (a) except for RAD26.Same as (a) except for RAD51.Same as (a) except for RTEL1.
(TIF)

S9 Fig Phylogenetic relationships among spirotrich ciliates. Maximum likelihood phylogenetic tree based on **small-subunit ribosomal RNA**.
(TIF)

S10 Fig Phylogenetic trees for RNA polymerase II factors. Maximum likelihood phylogenetic tree of RPB2. The grey and blue boxes indicate predicted RPB2a and RPB2b paralogs, respectively.Same as (a) except for RPB4.Same as (a) except for RPB7.Same as (a) except for SEN1.Same as (a) except for ELF1.Same as (a) except for RTT103.Same as (a) except for SPT5.
(TIF)

S11 Fig Phylogenetic trees for DNA recombination factors. Maximum likelihood phylogenetic tree of MCM8. The grey and blue boxes indicate predicted MCM8a/b and MCM8c paralogs, respectively.Same as (a) except for MCM9.Same as (a) except for HOP2.Same as (a) except for MND1.Same as (a) except for BMI1.Same as (a) except for RAD51.
(TIF)

S12 Fig Expression profiles of ciliate macronuclear development-specific orthogroup members across human body sites. Heatmap representation of relative mRNA expression levels across human body sites for a set of mRNAs preferentially expressed in EBV transformed lymphocytes.Same as (a) except for mRNAs preferentially expressed in testis.
(TIF)

S13 Fig Putative DNA and RNA regulatory elements that specify mRNA expression profiles during macronuclear development. The leftmost column shows the consensus sequence of significant scoring motifs, location indicates whether the motif was discovered upstream (5’) or downstream (3’) of coding sequences, p-values came from HOMER.(left) Motif1 tends to occur closer to the adjacent coding sequence in mRNAs in module 4 compared to control mRNAs. (right) The top scoring motif from orthologous sequences in *S*. *histriomuscorum*.Same as b, except for motif2.
(TIF)

S14 Fig The micronuclear genome contributes hundreds of genes to the macronuclear development expression program. Heatmap representation of relative mRNA expression of 259 mRNAs derived from micronuclear genome encoded genes during macronuclear development. mRNAs are organized by hierarchical clustering. Relative mRNA expression was normalized such that log_2_(TPM +1) levels in 0 hr cells was zero on average.Barplot showing domains that were most often found in the protein products of the 259 mRNAs in (a).
(TIF)

S1 Table Comparision of the number of RNA polymerase II subunits encoded by *Oxytricha* and *Arabidopsis* respectively. (TIF)

S1 Text Conserved germline factors involved in RNA metabolism, chromatin modification and SUMOlyation. (PDF)

S2 Text Protein domains and potential functions of recently acquired RNA and DNA-binding proteins with potential roles in macronuclear development. (PDF)

S3 Text Further characterization of two putative regulatory elements. (PDF)

S1 Dataset Normalized RNA expression data (FPKM) across the fourteen samples. (TXT)

S2 Dataset Orthofinder results. Column 1 = orthogroup, column 2 = gene id, column 3 = species, column 4 = annotations as described in Materials and Methods.
(ZIP)

S3 Dataset Assigned gene names. Column 1 = gene id, column 2 = gene name, column 3 = orthogroup, column 4 = gene description/annotation, column 5–10 = member of various curated gene lists (1 = yes), column 11 = associated curated gene list used for Figs.
(TXT)

S4 Dataset mRNAs preferentially expressed during macronuclear development. Organized by membership in modules 1–6 as noted in column 3.
(TXT)

S5 Dataset Orthogroup members preferentially expressed during macronuclear development in *Oxytricha* and *Tetrahymena* or *Paramecium*. The first table is *Oxytricha*, followed by *Tetrahymena*, then *Paramecium*. Column 1 = gene id, column 2 = annotation, column 3 = orthogroup, column 4 -… = normalized mRNA expression results.
(XLSX)

## Abbreviations

dsRBD: double-stranded RNA-binding domain
HMG: High mobility group
IES: Internally eliminated sequence
MAC: Macronucleus
MDS: Macronuclear destined sequence
MIC: Micronucleus
PARP: Poly(ADP-ribose) polymerase
RPA: Replication protein A
RRM: RNA-recognition motif
