# Supplementary figures and images for: Preferential expression of scores of functionally and evolutionarily diverse DNA and RNA-binding proteins during Oxytricha trifallax macronuclear development
Source: PLoS One. 2017 Feb 16;12(2):e0170870. doi: 10.1371/journal.pone.0170870 (PMC5312943; doi:10.1371/journal.pone.0170870)

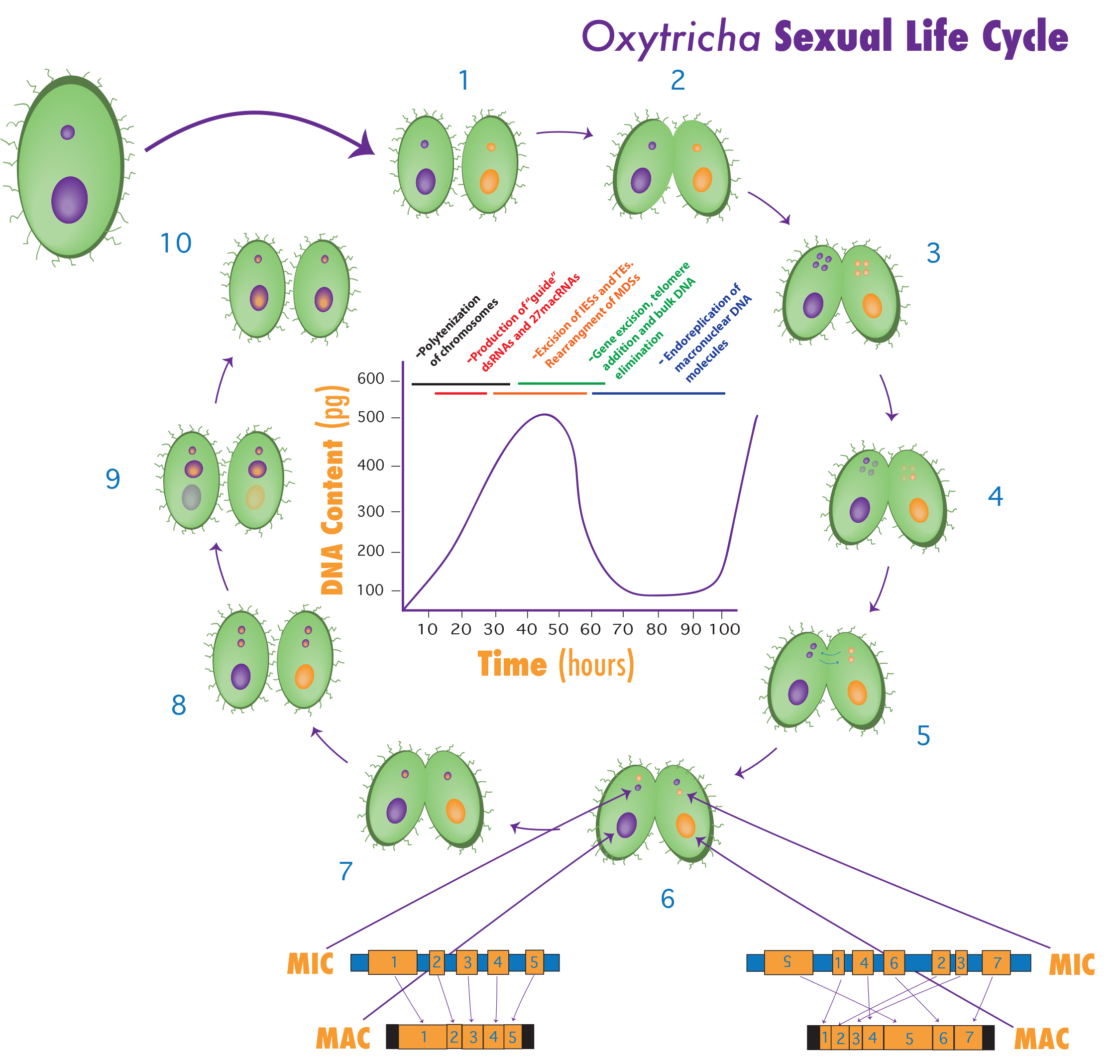

Supplement: S1 Fig — 1. Two vegetative O. trifallax cells of different mating types (represented by the difference in nuclei colors). 2. Under starvation conditions the two cells fuse and begin to conjugate. 3. A parental micronucleus in each cell undergoes meiosis. 4–6. Three of the newly formed haploid micronuclei will break down while the remaining one will undergo mitosis. One mitosis-derived haploid micronucleus is exchanged between the mating cells. 7. The newly acquired micronucleus fuses with the remaining maternal micronucleus to become diploid. 8. The newly formed diploid micronucleus undergoes mitosis. 9. One newly formed micronucleus develops into a new macronucleus while the maternal macronucleus is broken down and degraded. 10. Two genetically identical exconjugant O. trifallax cells. Inside the circle is a graph showing the general timing of macronuclear development events in hours and the corresponding DNA content of the developing macronucleus. Outside of the circle are the alternate microculear and macronuclear versions of two hypothetical genes, one scrambled and one nonscrambled (MDSs in orange, IESs and nongenic DNA in blue and telomeres in black) [20, 29, 31] (TIF) [file pone.0170870.s001.tif]

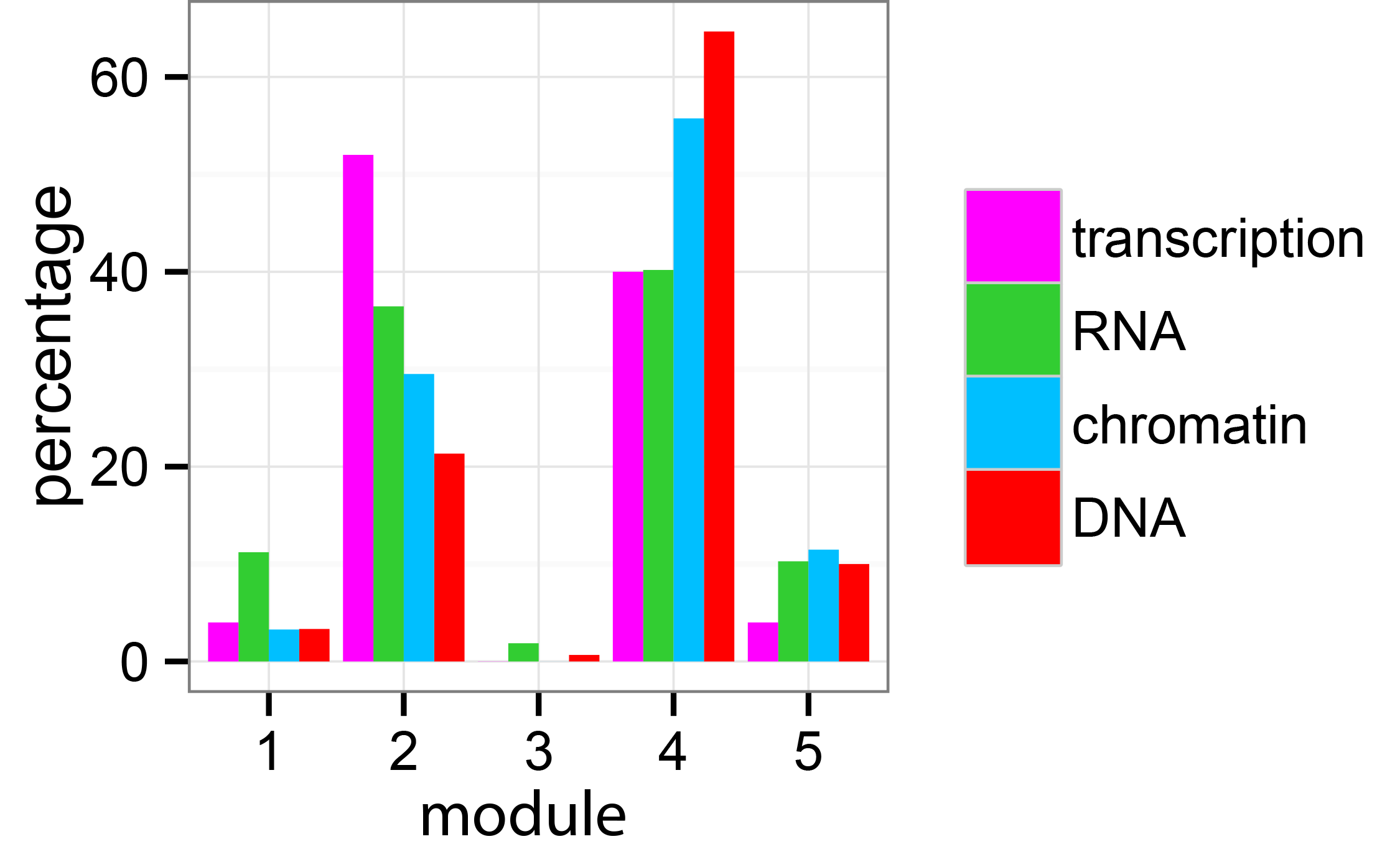

Supplement: S2 Fig — Barplot representation of the percentage of macronuclear development-specific members of each of the four gene lists in modules 1–5. (TIF) [file pone.0170870.s002.tif]

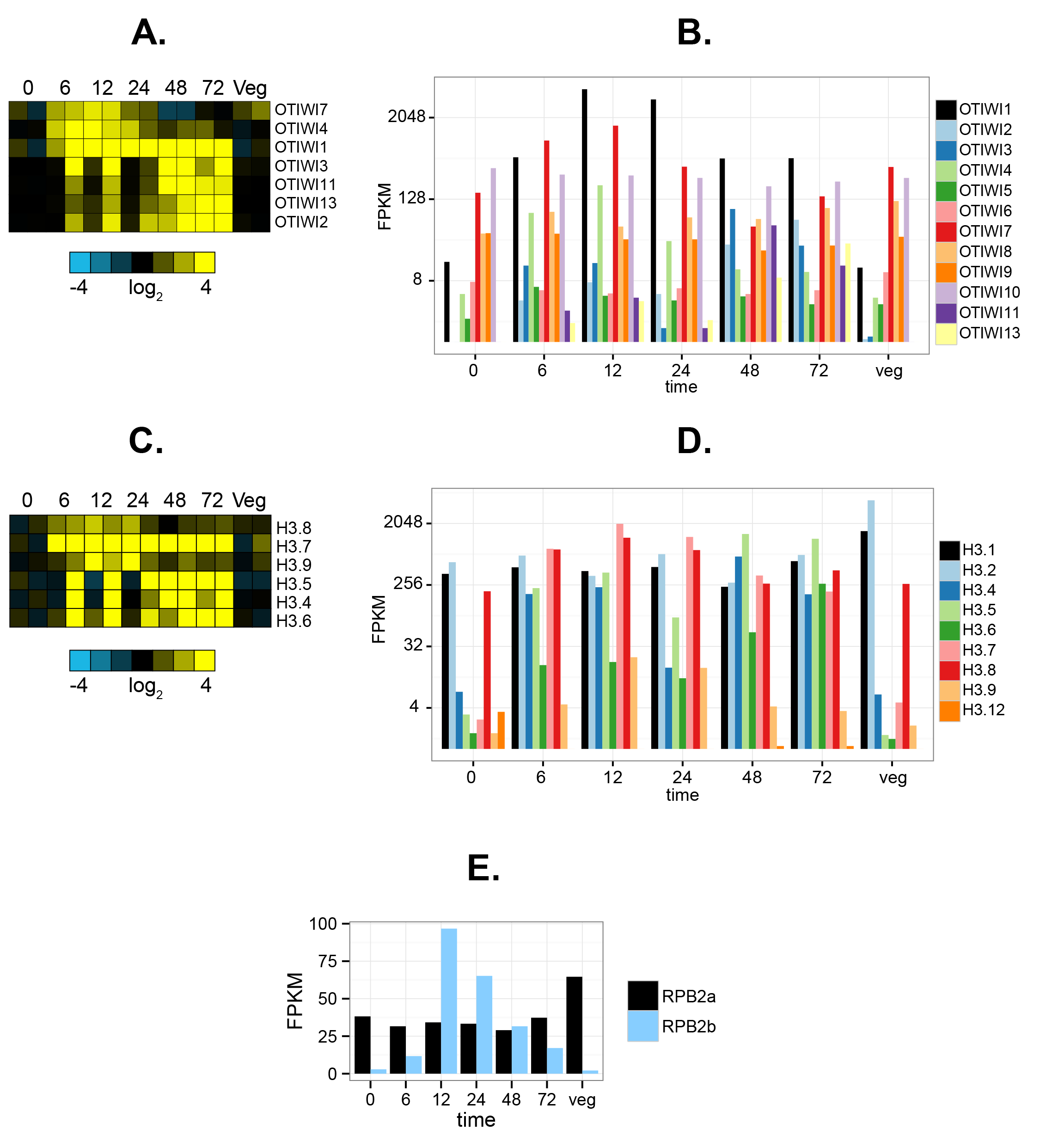

Supplement: S3 Fig — Heatmap representation of mRNA expression profiles of PIWI paralogs preferentially expressed during macronuclear development.Barplot representation of estimated absolute RNA expression levels (FPKM) of PIWI paralogs.Same as (a) except for Histone H3.Same as (b) except for Histone H3.Same as (b) except for RPB2. (TIF) [file pone.0170870.s003.tif]

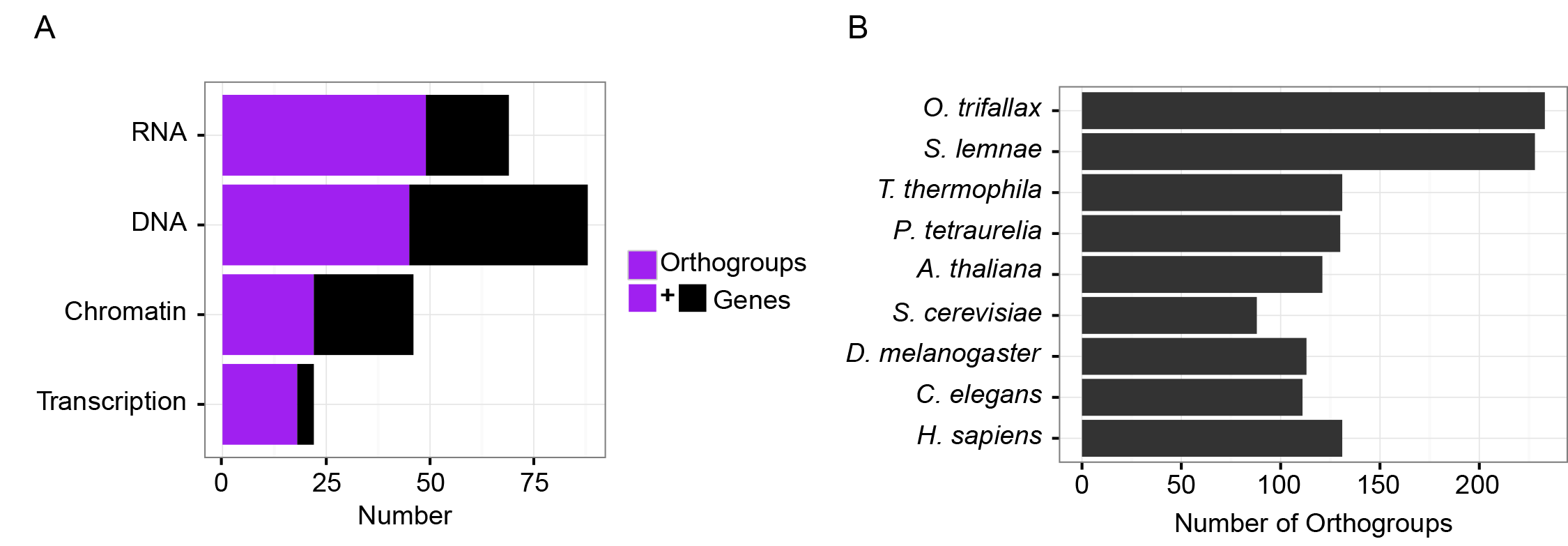

Supplement: S4 Fig — Barplot representation of the number of orthogroups (purple) and genes (purple + black) from the orthogroups identified in (b) that were associated with four manually curated gene lists associated with RNA and DNA biologyBarplot representation showing orthogroups containing at least two members in Oyxtricha in which at least one member is preferentially expressed during macronuclear development; for the other species analyzed, the barplot shows the number of those orthogroups with at least one member in said species. (TIF) [file pone.0170870.s004.tif]

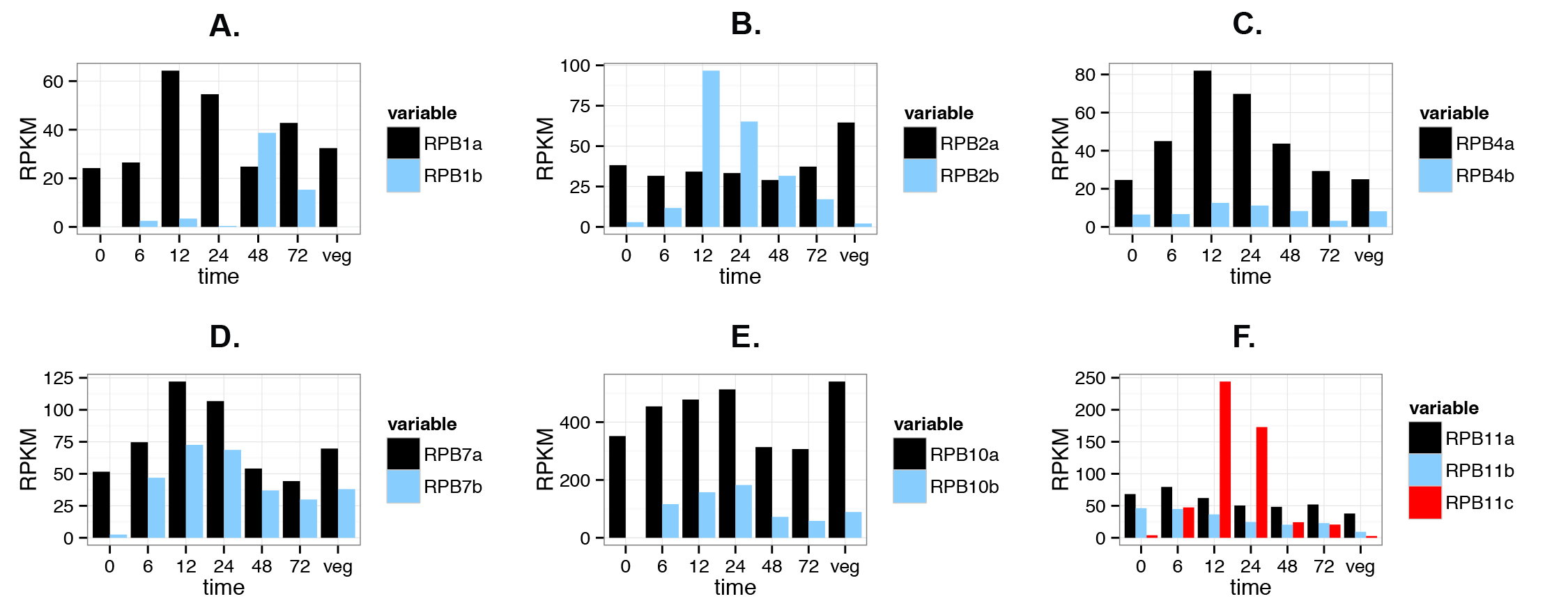

Supplement: S5 Fig — Barplot representation of estimated absolute RNA expression levels (FPKM) of RPB1 paralogs during macronuclear development.Same as (a) except for RPB2.Same as (a) except for RPB4.Same as (a) except for RPB7.Same as (a) except for RPB10.Same as (a) except for RPB11. (TIF) [file pone.0170870.s005.tif]

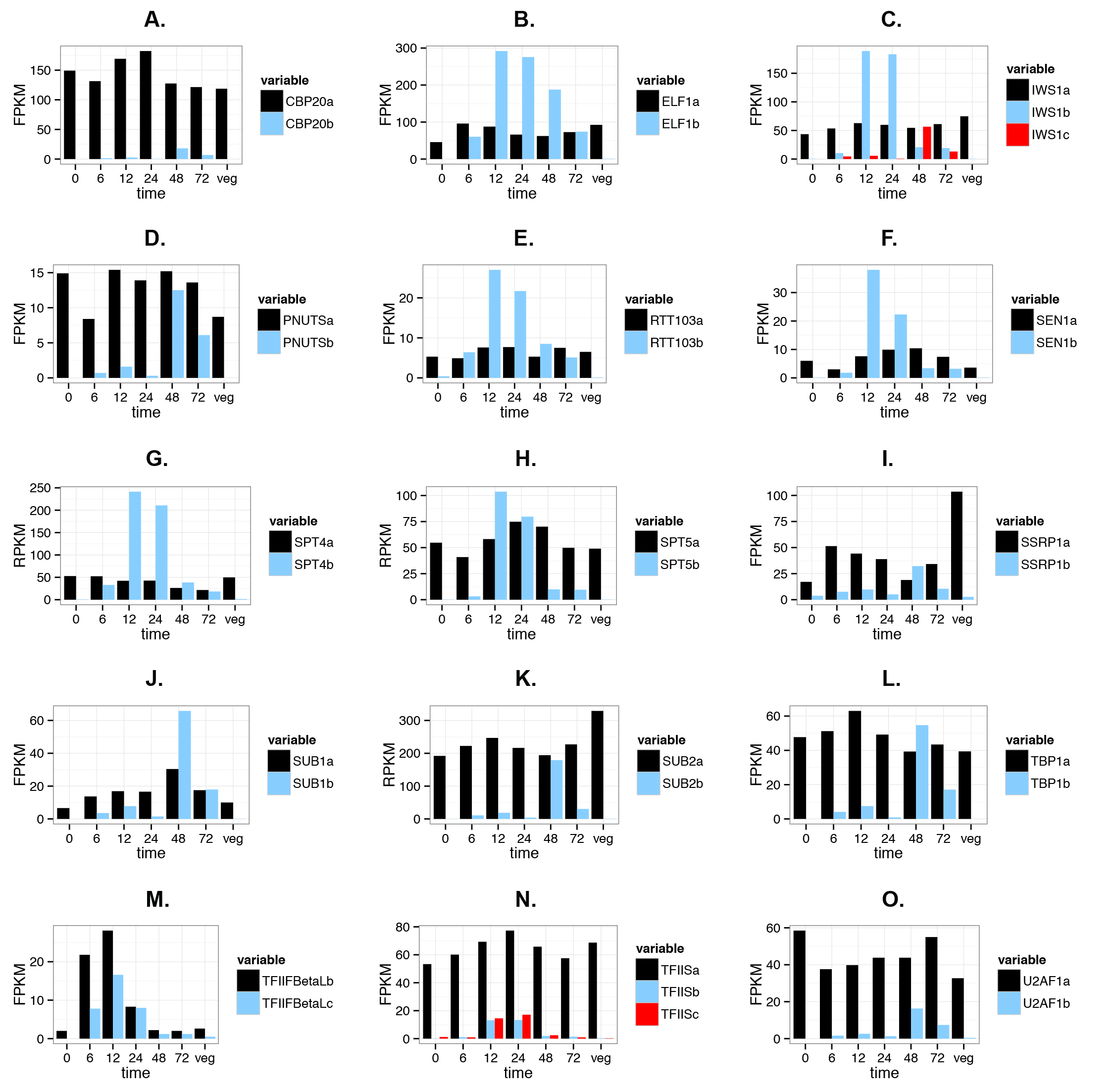

Supplement: S6 Fig — Barplot representation of estimated absolute RNA expression levels (FPKM) of CBP20 paralogs expressed during macronuclear development.Same as (a) except for ELF1.Same as (a) except for IWS1.Same as (a) except for PNUTS.Same as (a) except for RTT103.Same as (a) except for SEN1.Same as (a) except for SPT4.Same as (a) except for SPT5.Same as (a) except for SSRP1.Same as (a) except for SUB1.Same as (a) except for SUB2.Same as (a) except for TBP1.Same as (a) except for TFIIFBeta.Same as (a) except for TFIIS.Same as (a) except for U2AF1. (TIF) [file pone.0170870.s006.tif]

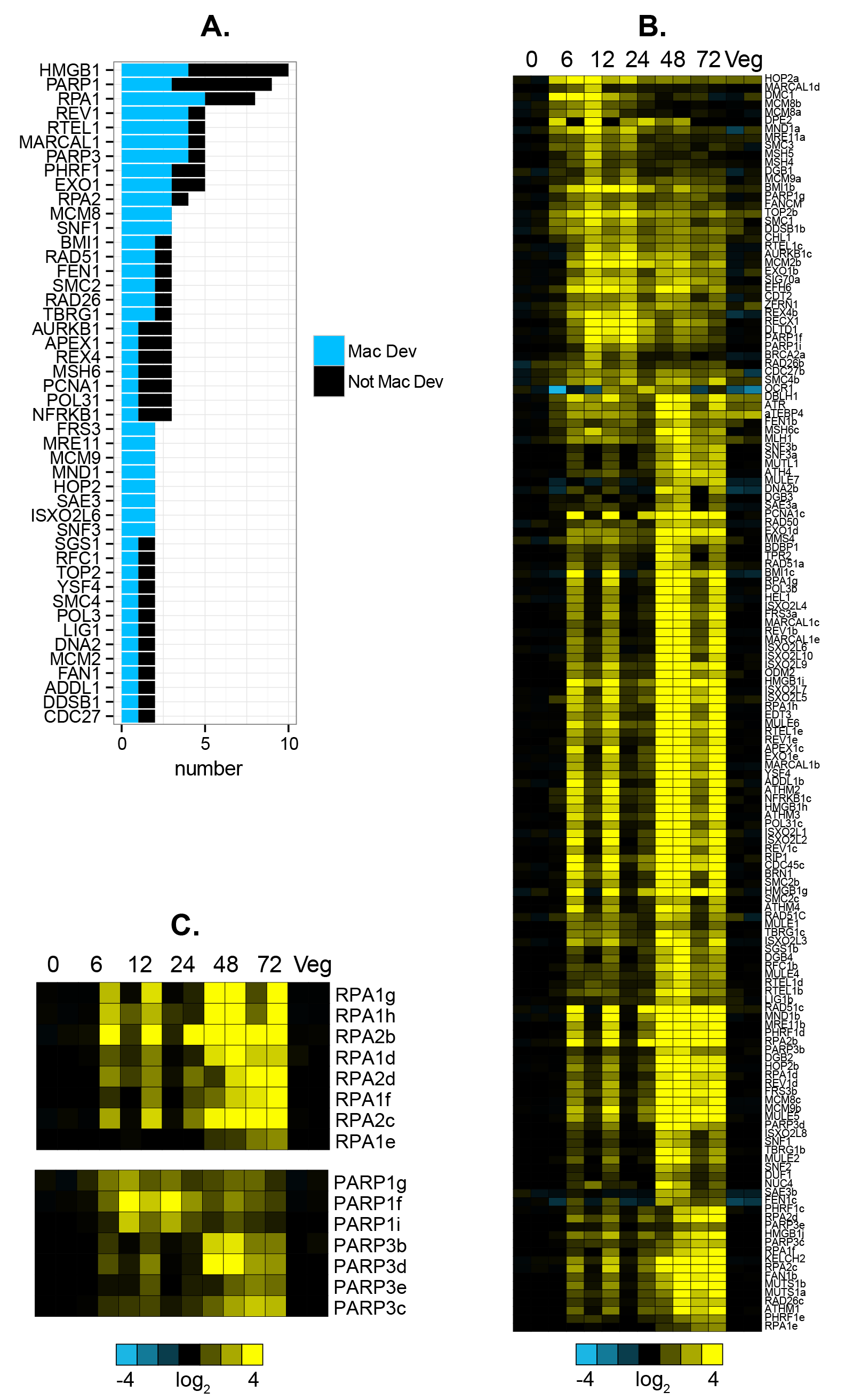

Supplement: S7 Fig — Barplot showing DNA synthesis, repair and recombination genes with multiple members in O. trifallax in which at least one member is preferentially expressed during macronuclear development. Blue shows the number that are preferentially expressed during macronuclear development, black shows the number that are not preferentially expressed during macronuclear development.Heatmap representation of mRNA expression profiles of DNA synthesis, repair and recombination genes preferentially expressed during macronuclear development.Heatmap representation of mRNA expression profiles of RPA and PARP gene family members preferentially expressed during macronuclear development. (TIF) [file pone.0170870.s007.tif]

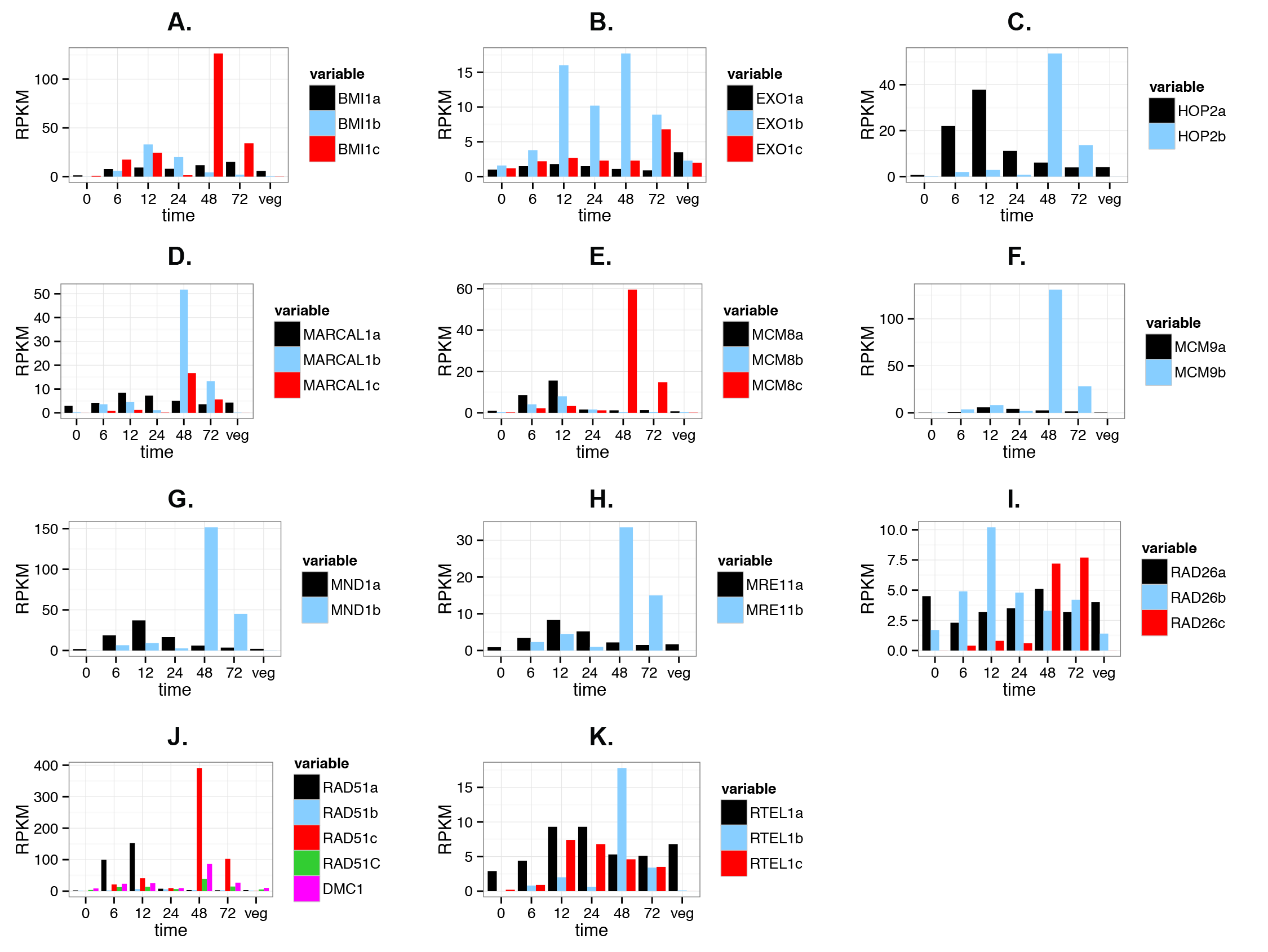

Supplement: S8 Fig — Barplot representation of estimated absolute RNA expression levels (FPKM) of BMI paralogs during macronuclear development.Same as (a) except for EXO1.Same as (a) except for HOP2.Same as (a) except for MARCAL1.Same as (a) except for MCM8.Same as (a) except for MCM9.Same as (a) except for MDN1.Same as (a) except for MRE11.Same as (a) except for RAD26.Same as (a) except for RAD51.Same as (a) except for RTEL1. (TIF) [file pone.0170870.s008.tif]

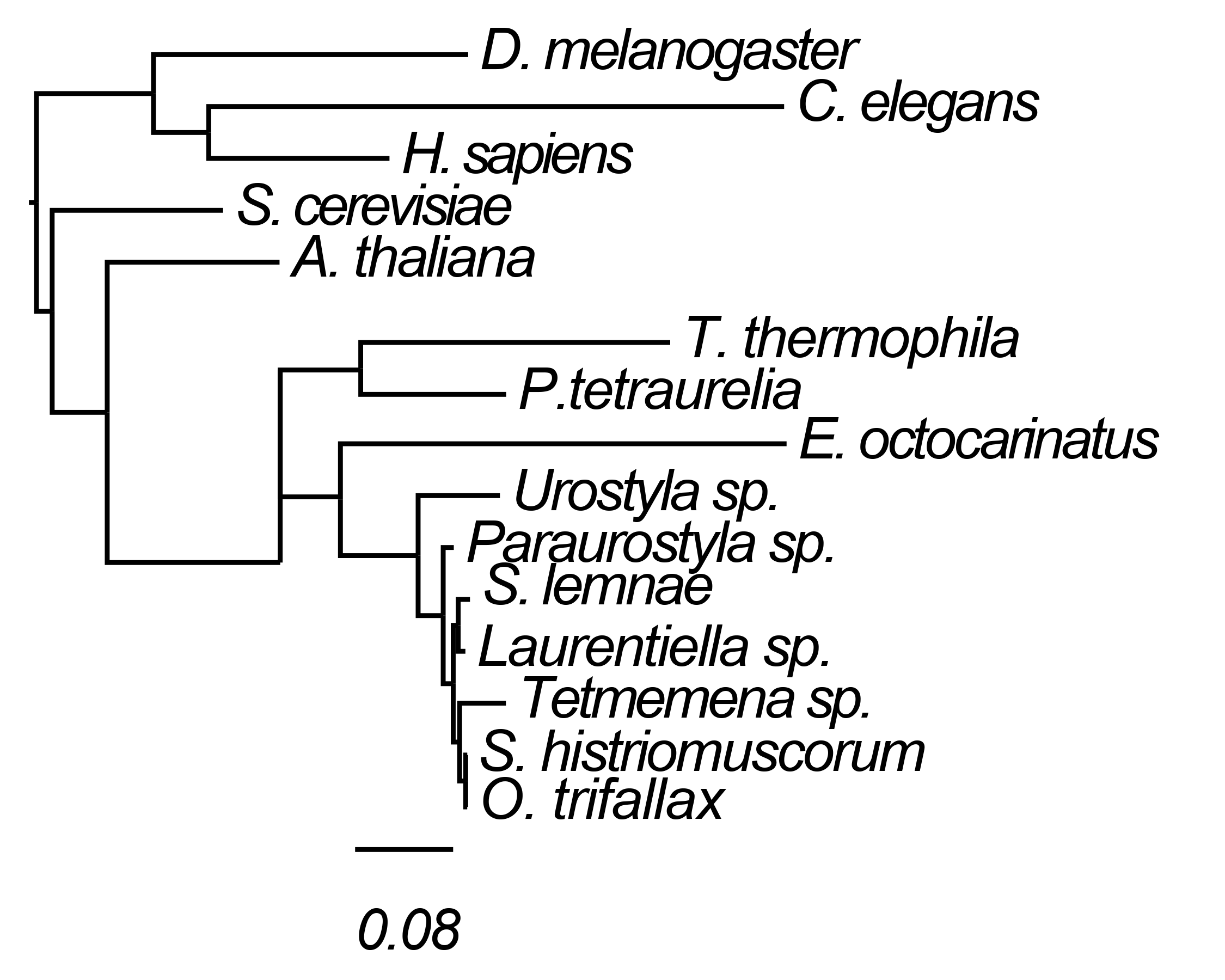

Supplement: S9 Fig — Maximum likelihood phylogenetic tree based on small-subunit ribosomal RNA. (TIF) [file pone.0170870.s009.tif]

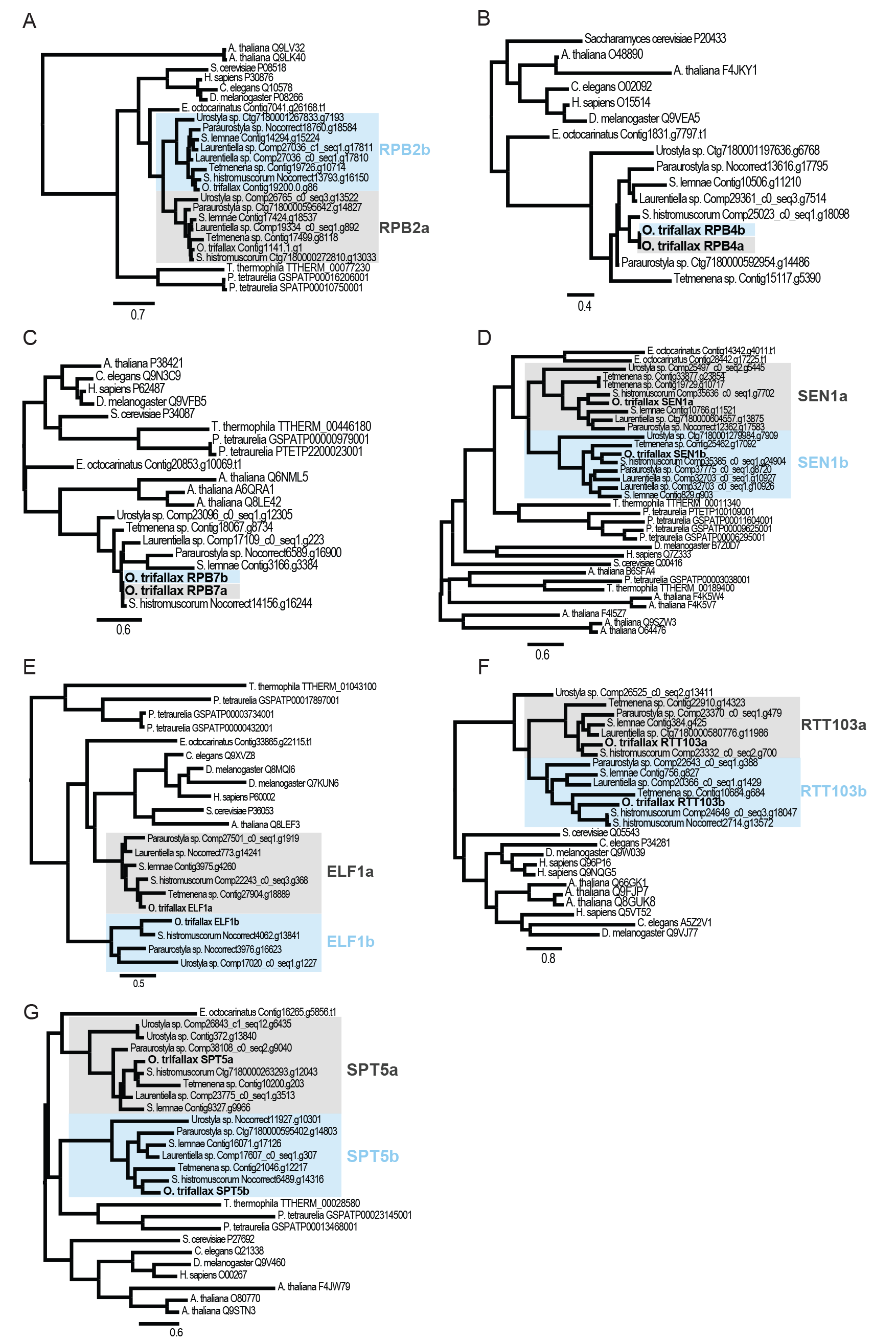

Supplement: S10 Fig — Maximum likelihood phylogenetic tree of RPB2. The grey and blue boxes indicate predicted RPB2a and RPB2b paralogs, respectively.Same as (a) except for RPB4.Same as (a) except for RPB7.Same as (a) except for SEN1.Same as (a) except for ELF1.Same as (a) except for RTT103.Same as (a) except for SPT5. (TIF) [file pone.0170870.s010.tif]

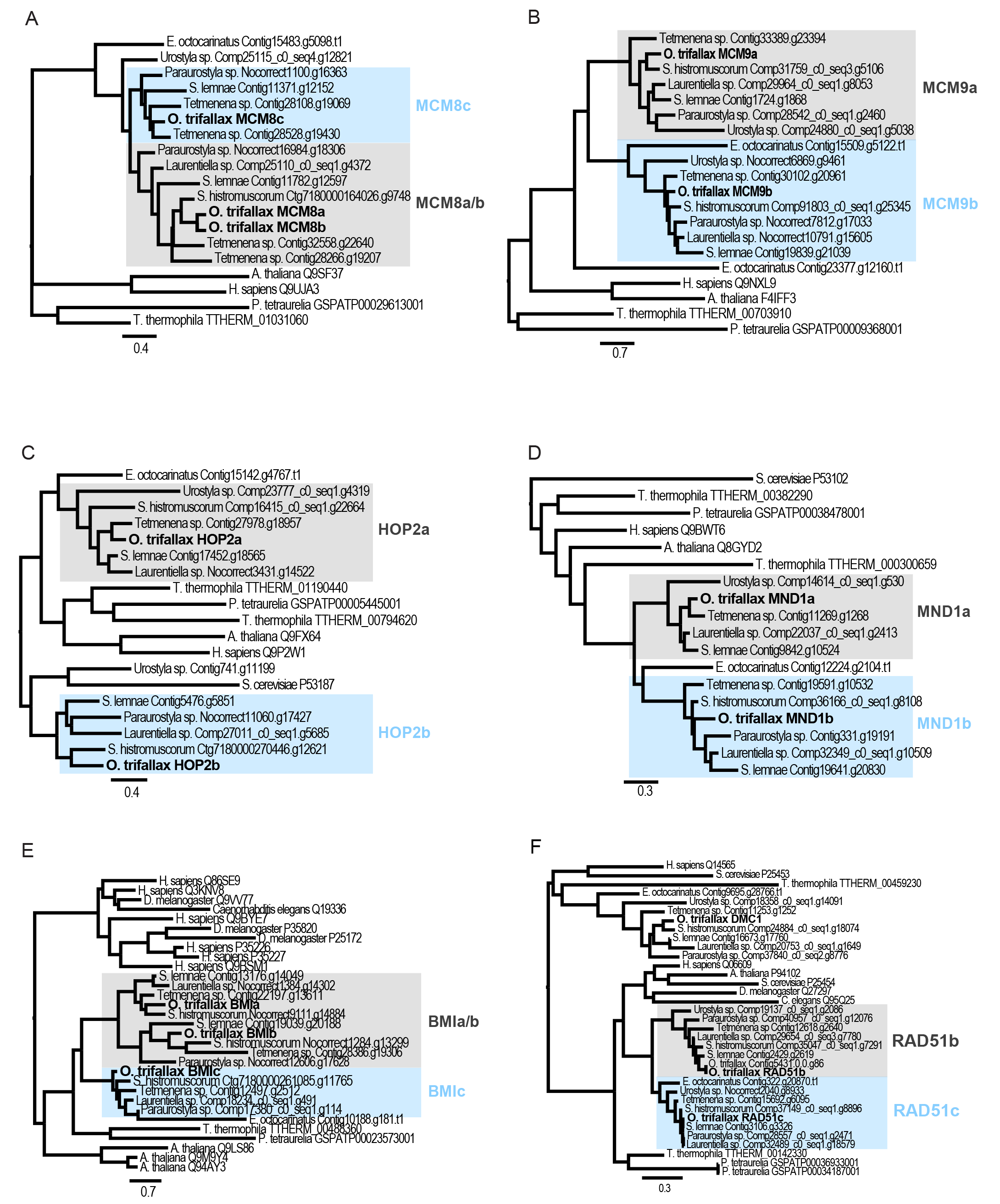

Supplement: S11 Fig — Maximum likelihood phylogenetic tree of MCM8. The grey and blue boxes indicate predicted MCM8a/b and MCM8c paralogs, respectively.Same as (a) except for MCM9.Same as (a) except for HOP2.Same as (a) except for MND1.Same as (a) except for BMI1.Same as (a) except for RAD51. (TIF) [file pone.0170870.s011.tif]

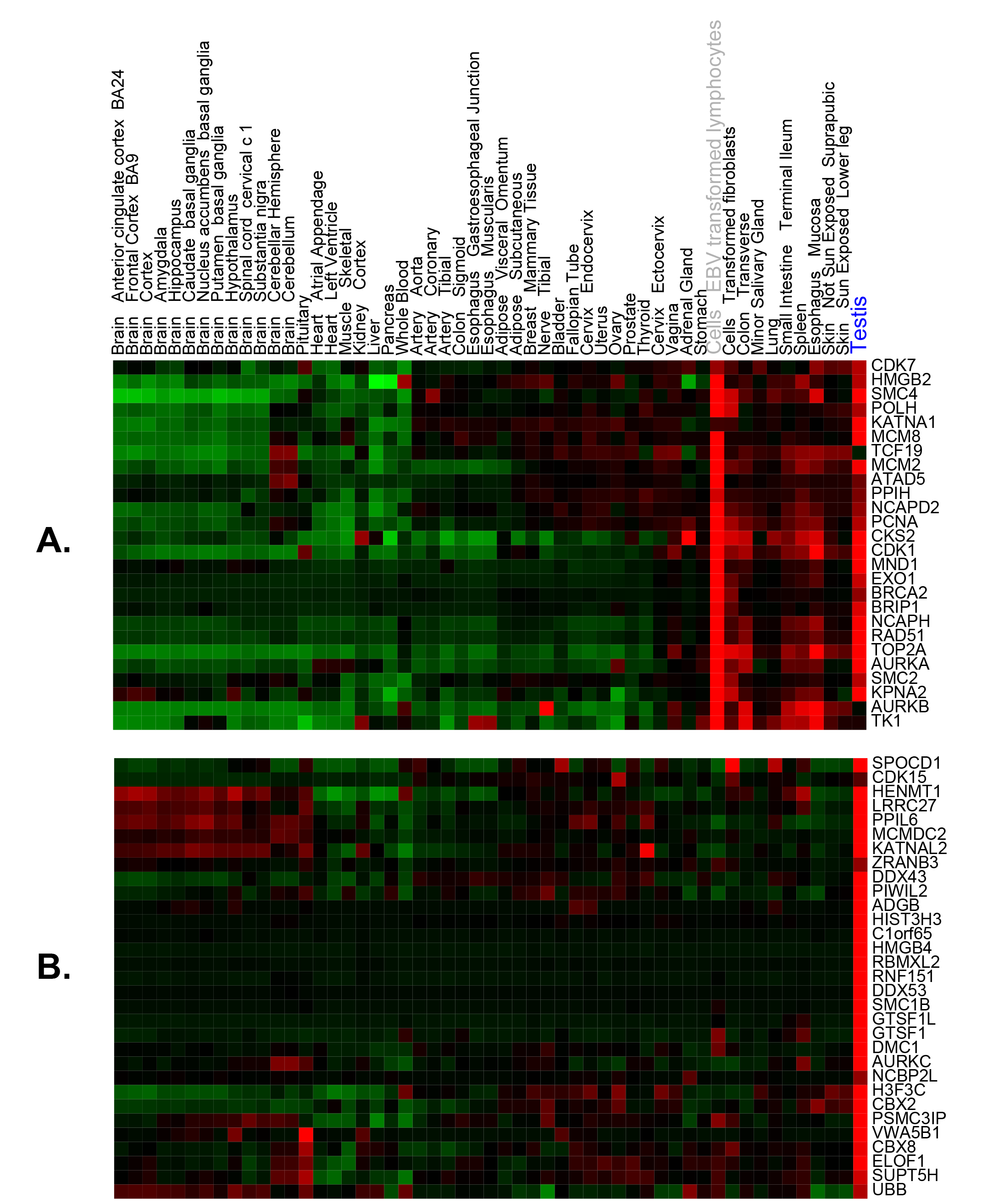

Supplement: S12 Fig — Heatmap representation of relative mRNA expression levels across human body sites for a set of mRNAs preferentially expressed in EBV transformed lymphocytes.Same as (a) except for mRNAs preferentially expressed in testis. (TIF) [file pone.0170870.s012.tif]

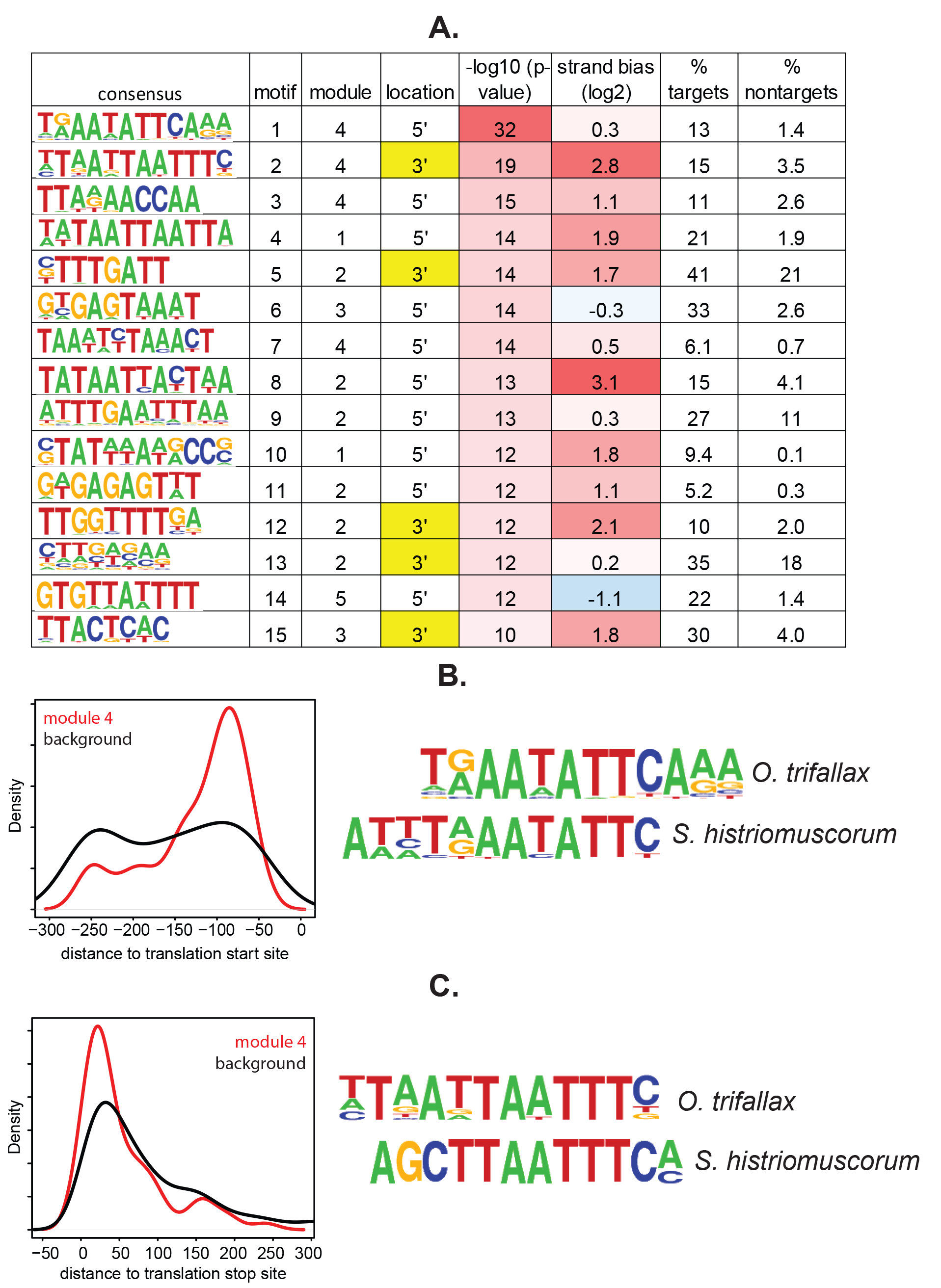

Supplement: S13 Fig — The leftmost column shows the consensus sequence of significant scoring motifs, location indicates whether the motif was discovered upstream (5’) or downstream (3’) of coding sequences, p-values came from HOMER.(left) Motif1 tends to occur closer to the adjacent coding sequence in mRNAs in module 4 compared to control mRNAs. (right) The top scoring motif from orthologous sequences in S. histriomuscorum.Same as b, except for motif2. (TIF) [file pone.0170870.s013.tif]

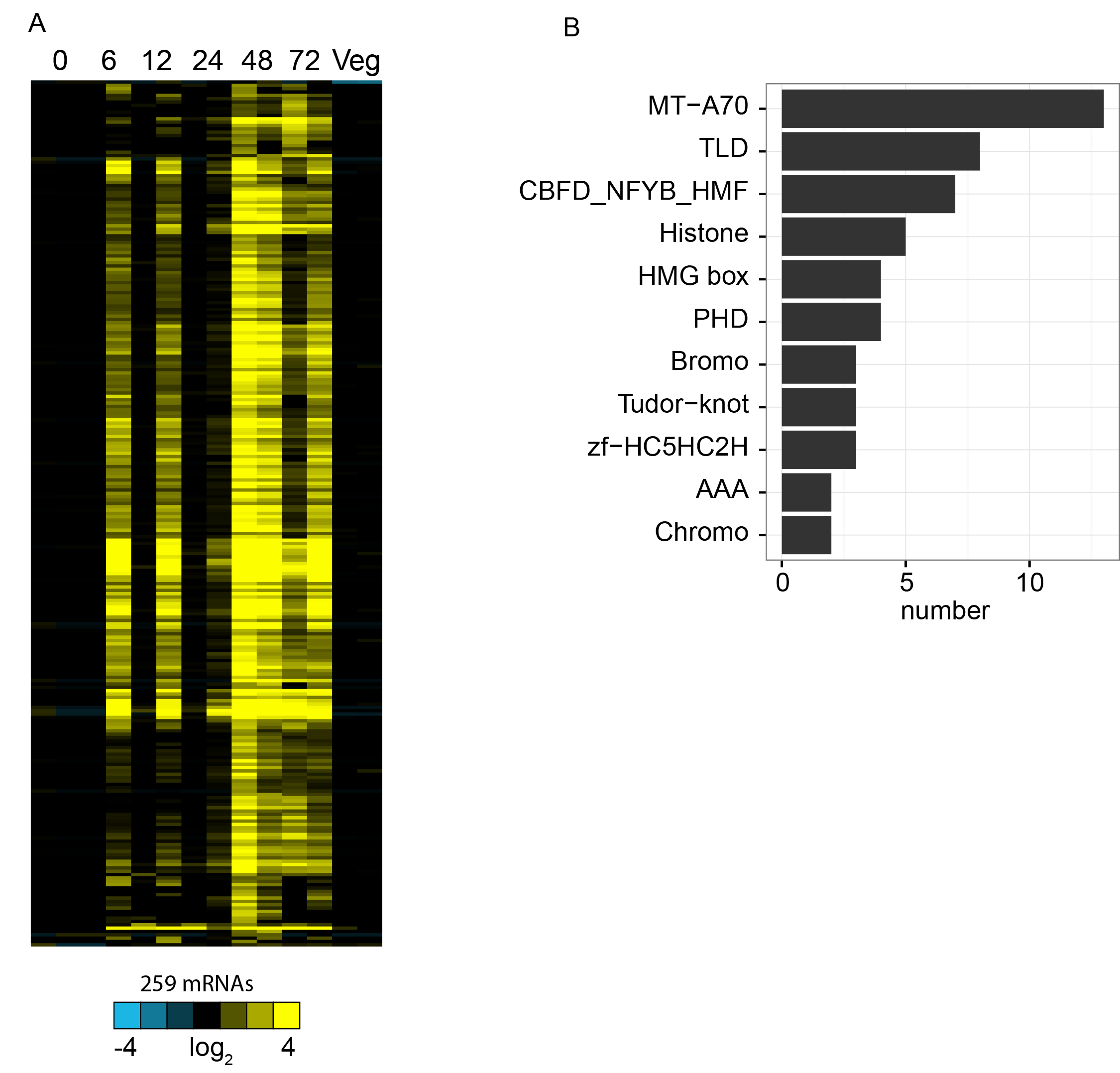

Supplement: S14 Fig — Heatmap representation of relative mRNA expression of 259 mRNAs derived from micronuclear genome encoded genes during macronuclear development. mRNAs are organized by hierarchical clustering. Relative mRNA expression was normalized such that log2(TPM +1) levels in 0 hr cells was zero on average.Barplot showing domains that were most often found in the protein products of the 259 mRNAs in (a). (TIF) [file pone.0170870.s014.tif]

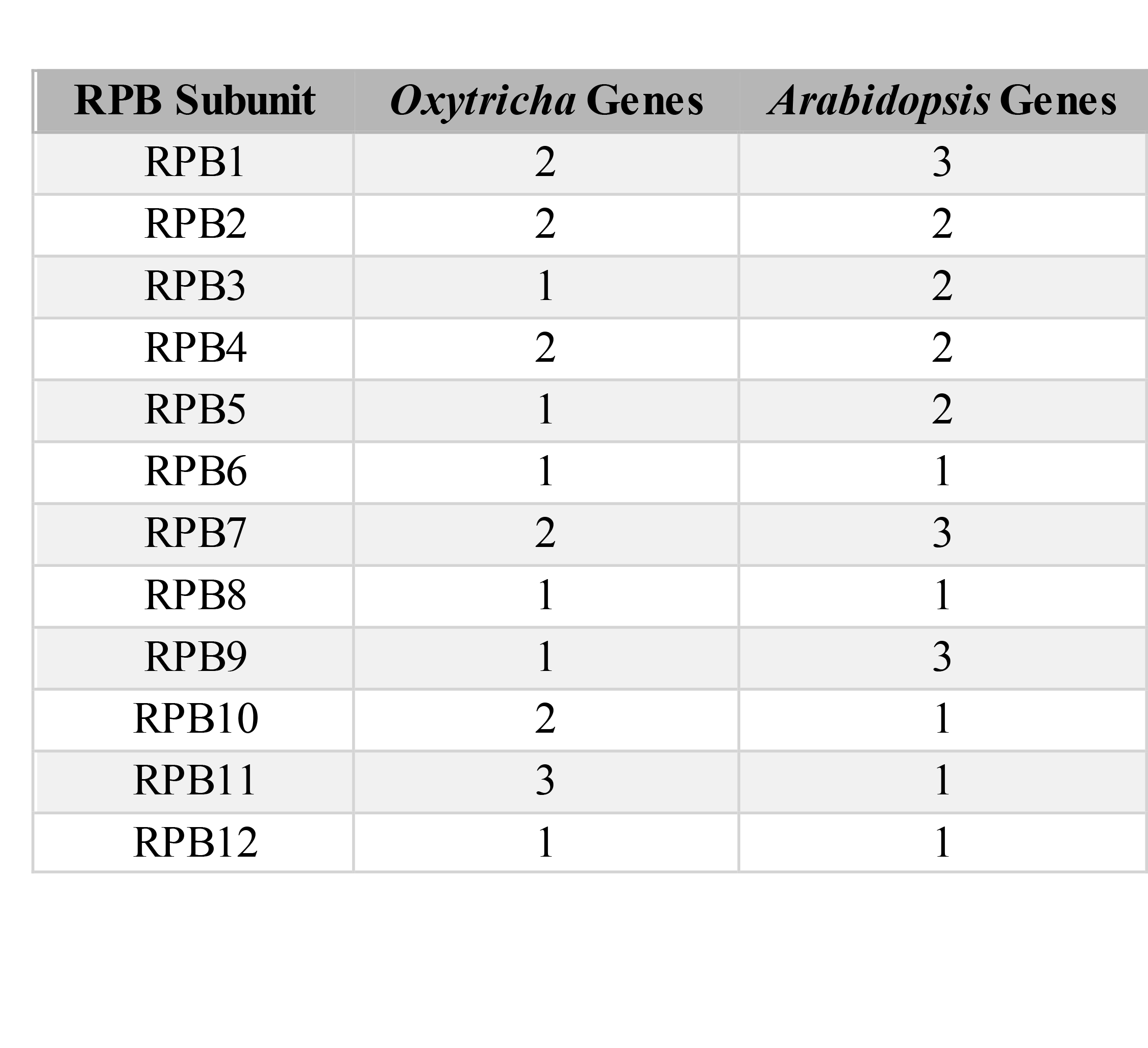

Supplement: S1 Table — (TIF) [file pone.0170870.s015.tif]
