# Supplementary material for: Preferential expression of scores of functionally and evolutionarily diverse DNA and RNA-binding proteins during Oxytricha trifallax macronuclear development
Source: PLoS One. 2017 Feb 16;12(2):e0170870. doi: 10.1371/journal.pone.0170870 (PMC5312943; doi:10.1371/journal.pone.0170870)
Supplement: S1 Text — (PDF) [file pone.0170870.s016.pdf]

Conserved Germline Factors Involved in RNA Metabolism, Chromatin Modification and SUMOlyation

RNA-binding proteins have diverse roles in germline processes in addition to the PIWI pathway [1]. For instance, *O. trifallax* BRUNO is homologous to RRM containing CELF/BRUNO family. *D. melanogaster* Bruno is preferentially expressed in ovaries and testis and regulates multiple mRNAs involved in female and male gametogenesis [2]. *A. thaliana* homolog FCA is a master regulator of flower development [3]. DDX43b is a DEAD box RNA helicases that is homologous to DDX5/17/43/53 in humans and macronuclear anlagen localized LIA2 in *T. thermophila* [4]. *O. trifallax* Mex1f is homologous to *C. elegans* Mex1/5/6 CCCH-type zinc-finger proteins which are required maternally for segregation of P granules, germ cell formation and somatic cell differentiation in the early embryo [5, 6]. *O. trifallax* ML1b is homologous to MEI2 in fission yeast, which is a master regulator of meiosis [7]; ML1-5 in *A. thaliana* are concentrated in young buds and reproductive organ primordia [8]. *O. trifallax* IME4c belongs to the MT-A70-like family and is likely homologous to budding yeast IME4, which is an m6A RNA methyltransferase required for entry into meiosis [9]. IME4c expression peaks early during macronuclear development while demethylase ALKBH5b peaks late, suggesting dynamic regulation of mRNA m6A methylation and demethylation during the developmental program. *O. trifallax* EMA1 is a DEAD box helicase homologous to *T. thermophila* EMA1, which is implicated in small RNA-RNA/DNA pairing during macronuclear development [10]. *O. trifallax* EMA1 expression correlates with that of macronuclear development specific DICER and RDRP, and could be part of PIWI targeting complexes in *O. trifallax*, as suggested for its *T. thermophila* ortholog.

Dynamic chromatin modifications and restructuring are hallmarks of germline developmental programs with seminal discoveries made in *Tetrahymena*. For instance, *T. thermophila* Pdd1 is a HP1-family

chromodomain protein specifically expressed during macronuclear development that associates with DNA destined for elimination via binding methylated lysine-9 residues of histone H3 [11]. *O. trifallax* Pdd1 homologs HP1b/c expression levels peak 72 hrs into macronuclear development, coincident with elimination of ~90% of genome (Figure 7E). It is possible HP1b/c help mark non-genic regions for DNA elimination in the developing macronucleus. *O. trifallax* BRD1c is a bromo and BET domain-containing protein with sequence similarity to testis-specific BRDT in humans and *Arabidopsis* GTE1, which plays a role in seed germination [12]. Human BRDT specifically binds acetylated histone H4 and is required for chromocenter organization in sperm, a structure comprised of peri-centromeric heterochromatin [13, 14]. BRD1c expression peaks at 48 hrs. *O. trifallax* TCF19 is a BAH and PHD domain containing protein homologous to SHL1 in *A. thaliana*, which represses floral initiation via binding lysine 4 di- and trimethylated histone H3 [15]. *O. trifallax* HMGB1g-j are homologous to human HMGB1, which bends DNA to facilitate V(D)J recombination and assembly of nucleosomes on chromatin [16, 17]. HMGB1g-j's expression levels peak at 48 hours and these proteins could play roles facilitating DNA looping during IES excision and gene unscrambling. *O. trifallax* ANP32c-f are leucine rich repeat proteins homologous to ANP32 family of histone chaperones. Human Anp32e removes H2A.Z from DNA double-strand breaks and promotes nucleosome reorganization and DNA repair [18-20]. *O. trifallax* SMARCA1c is homologous to SWI2/SNF2 family of ATPase subunits of nucleosome remodeling complexes. During spermatogenesis in *D. melanogaster* ISWI promotes chromatin condensation and then at fertilization facilitates chromatin decondensation [21]. *O. trifallax* CHD1b is homologous to CHD1 ATP-dependent chromatin-remodeling factor which functions as the substrate recognition component of the SAGA complex. CHD1 is required for deposition of germline specific Histone H3 into chromatin in *D. melanogaster* [22].

SUMOylation and ubquitination are protein modifications associated with an array of processes, including regulation of cell division, DNA repair and recombination [23, 24]. In *P. tetraurelia* and *T. thermophila*, SUMOylation increases dramatically during macronuclear development and genes

encoding proteins in the SUMOylation pathway are required for this process [25, 26]. Our set of conserved macronuclear development genes includes homologs of SUMO (SUMO1b), E1 (AOS1 and UBA2), E2 (UBC9) and E3 SUMO ligases (PHRF1c-e), ULP1 SUMO peptidase (ULP1b) as well as ubiquitin (UBCL1a)(Figure 7F). Thus, in addition to poly ADP-ribosylation, SUMOylation and ubiquitination appear to play important regulatory roles during macronuclear development in *O. trifallax*.

1. Lehmann R: **Germline stem cells: origin and destiny**. *Cell Stem Cell* 2012, **10**(6):729-739.
2. Chekulaeva M, Hentze MW, Ephrussi A: **Bruno acts as a dual repressor of oskar translation, promoting mRNA oligomerization and formation of silencing particles**. *Cell* 2006, **124**(3):521-533.
3. Macknight R, Bancroft I, Page T, Lister C, Schmidt R, Love K, Westphal L, Murphy G, Sherson S, Cobbett C *et al*: **FCA, a gene controlling flowering time in Arabidopsis, encodes a protein containing RNA-binding domains**. *Cell* 1997, **89**(5):737-745.
4. Yao MC, Yao CH, Halasz LM, Fuller P, Rexer CH, Wang SH, Jain R, Coyne RS, Chalker DL: **Identification of novel chromatin-associated proteins involved in programmed genome rearrangements in Tetrahymena**. *J Cell Sci* 2007, **120**(Pt 12):1978-1989.
5. Guedes S, Priess JR: **The C. elegans MEX-1 protein is present in germline blastomeres and is a P granule component**. *Development* 1997, **124**(3):731-739.
6. Schubert CM, Lin R, de Vries CJ, Plasterk RH, Priess JR: **MEX-5 and MEX-6 function to establish soma/germline asymmetry in early C. elegans embryos**. *Mol Cell* 2000, **5**(4):671-682.
7. Watanabe Y, Yamamoto M: **S. pombe mei2+ encodes an RNA-binding protein essential for premeiotic DNA synthesis and meiosis I, which cooperates with a novel RNA species meiRNA**. *Cell* 1994, **78**(3):487-498.
8. Kaur J, Sebastian J, Siddiqi I: **The Arabidopsis-mei2-like genes play a role in meiosis and vegetative growth in Arabidopsis**. *Plant Cell* 2006, **18**(3):545-559.
9. Shah JC, Clancy MJ: **IME4, a gene that mediates MAT and nutritional control of meiosis in Saccharomyces cerevisiae**. *Mol Cell Biol* 1992, **12**(3):1078-1086.
10. Aronica L, Bednenko J, Noto T, DeSouza LV, Siu KW, Loidl J, Pearlman RE, Gorovsky MA, Mochizuki K: **Study of an RNA helicase implicates small RNA-noncoding RNA interactions in programmed DNA elimination in Tetrahymena**. *Genes Dev* 2008, **22**(16):2228-2241.
11. Liu Y, Mochizuki K, Gorovsky MA: **Histone H3 lysine 9 methylation is required for DNA elimination in developing macronuclei in Tetrahymena**. *Proc Natl Acad Sci U S A* 2004, **101**(6):1679-1684.
12. Duque P, Chua NH: **IMB1, a bromodomain protein induced during seed imbibition, regulates ABA- and phyA-mediated responses of germination in Arabidopsis**. *Plant J* 2003, **35**(6):787-799.
13. Berkovits BD, Wolgemuth DJ: **The role of the double bromodomain-containing BET genes during mammalian spermatogenesis**. *Current topics in developmental biology* 2013, **102**:293-326.
14. Gaucher J, Boussouar F, Montellier E, Curtet S, Buchou T, Bertrand S, Hery P, Jounier S, Depaux A, Vitte AL *et al*: **Bromodomain-dependent stage-specific male genome programming by Brdt**. *EMBO J* 2012, **31**(19):3809-3820.
15. Lopez-Gonzalez L, Mouriz A, Narro-Diego L, Bustos R, Martinez-Zapater JM, Jarillo JA, Pineiro M: **Chromatin-dependent repression of the Arabidopsis floral integrator genes involves plant specific PHD-containing proteins**. *Plant Cell* 2014, **26**(10):3922-3938.
16. Stros M: **HMGB proteins: interactions with DNA and chromatin**. *Biochim Biophys Acta* 2010, **1799**(1-2):101-113.
17. Gellert M: **V(D)J recombination: RAG proteins, repair factors, and regulation**. *Annu Rev Biochem* 2002, **71**:101-132.
18. Obri A, Ouararhni K, Papin C, Diebold ML, Padmanabhan K, Marek M, Stoll I, Roy L, Reilly PT, Mak TW *et al*: **ANP32E is a histone chaperone that removes H2A.Z from chromatin**. *Nature* 2014, **505**(7485):648-653.

19. Mao Z, Pan L, Wang W, Sun J, Shan S, Dong Q, Liang X, Dai L, Ding X, Chen S *et al*: **Anp32e, a higher eukaryotic histone chaperone directs preferential recognition for H2A.Z**. *Cell Res* 2014, **24**(4):389-399.
20. Gursoy-Yuzugullu O, Ayrapetov MK, Price BD: **Histone chaperone Anp32e removes H2A.Z from DNA double-strand breaks and promotes nucleosome reorganization and DNA repair**. *Proc Natl Acad Sci U S A* 2015, **112**(24):7507-7512.
21. Doyen CM, Chalkley GE, Voets O, Bezstarosti K, Demmers JA, Moshkin YM, Verrijzer CP: **A Testis-Specific Chaperone and the Chromatin Remodeler ISWI Mediate Repackaging of the Paternal Genome**. *Cell reports* 2015, **13**(7):1310-1318.
22. Konev AY, Tribus M, Park SY, Podhraski V, Lim CY, Emelyanov AV, Vershilova E, Pirrotta V, Kadonaga JT, Lusser A *et al*: **CHD1 motor protein is required for deposition of histone variant H3.3 into chromatin in vivo**. *Science* 2007, **317**(5841):1087-1090.
23. Dantuma NP, van Attikum H: **Spatiotemporal regulation of posttranslational modifications in the DNA damage response**. *EMBO J* 2016, **35**(1):6-23.
24. Flotho A, Melchior F: **Sumoylation: a regulatory protein modification in health and disease**. *Annu Rev Biochem* 2013, **82**:357-385.
25. Matsuda A, Forney JD: **The SUMO pathway is developmentally regulated and required for programmed DNA elimination in Paramecium tetraurelia**. *Eukaryot Cell* 2006, **5**(5):806-815.
26. Nasir AM, Yang Q, Chalker DL, Forney JD: **SUMOylation is developmentally regulated and required for cell pairing during conjugation in Tetrahymena thermophila**. *Eukaryot Cell* 2015, **14**(2):170-181.
