# Supplementary material for: Preferential expression of scores of functionally and evolutionarily diverse DNA and RNA-binding proteins during Oxytricha trifallax macronuclear development
Source: PLoS One. 2017 Feb 16;12(2):e0170870. doi: 10.1371/journal.pone.0170870 (PMC5312943; doi:10.1371/journal.pone.0170870)
Supplement: S2 Text — (PDF) [file pone.0170870.s017.pdf]

Protein Domains and Potential Functions of Recently Acquired RNA and DNA-binding Proteins with Potential Roles in Macronuclear Development

We selected a subset of 33 of these genes based on their mRNA expression levels and predicted domains and looked more closely at their potential functions as well as their conservation across recently sequenced stichotrichs and *E. octocarinatus* (Figure 8B & C). Four genes in this group encode proteins with Alba nucleic-acid binding domains (ALBA 4-7). The Alba domain binds RNA and DNA with many eukaryotic genomes encoding at least one Alba domain protein in ribonuclease P/MRP subunit p20/p25 with occasional expansion of Alba domain proteins in specific lineages, for instance in *P. tetraurelia* and *Arabidopsis* [1]. The second gene recovered from a screen for macronuclear development-specific genes in *S. lemnae* was an Alba domain protein (MDP2) that was a founding member of a distinct clade on the Alba family tree[2]. *O. trifallax* encodes at least eleven Alba domain proteins. ALBA4-7 mRNA levels are extremely abundant and peak 12-24 hrs into macronuclear development coinciding with production of long dsRNA templates. Alba domain proteins with significant sequence identity are present in other stichotrichs and *E. octocarinatus*. Another protein that may interact with dsRNA templates is DSRBD1, which contains two double-stranded RNA-binding domains, is present in the other stichotrichs, and whose expression peaks at 12-24 hrs. KELCH115 is orthologous to MDP3 in *Stylonychia*, is extremely abundant, and is among eleven Kelch domain proteins preferentially expressed during macronuclear development. Kelch domain proteins are implicated in diverse processes, including DNA metabolism [3]. The founding member Kelch was identified as a female-sterile mutation that produced small, cuplike eggs in *Drosophila* [4].

Kelch participates in formation of ring canals that interconnect the oocyte and fifteen associated nurse cells. During macronuclear development in *Oxytricha* and other spirotrichs, proteinaceous “vesicles” transect and enclose regions of the polytene chromosomes in the developing macronucleus prior to bulk DNA elimination [5-8]. One potential role of Kelch proteins would be to participate in formation of these “vesicles”.

A number of genes encode proteins with domains implicated in DNA manipulation, and could therefore have roles in DNA rearrangements. ISXOL5 is one of ten ISXO2 transposase family members preferentially expressed during macronuclear development [9]. Their expression levels peak at 48 hrs and they are present in other stichotrichs except *Urostyla*, but are not seen elsewhere. MULE5 is among six genes encoding proteins containing the MULE transposase domain [9]. MULE expression levels peak at 48 hrs and these proteins are present in other stichotrichs and *E. octocarinatus*. Contig5440.0.g55 encodes a protein containing 3-5’ DNA exonuclease domain, expression peaks at 48-72 hrs, and the encoded protein is conserved in other stichotrichs and *E. octocarinatus*. Contig11653.0.g48 encodes a protein with an archaeal phage integrase domain, is conserved across stichotrichs and *E. octocarinatus* and its expression peaks at 48 hrs. Contig11874.0.g99 encodes a protein containing the Slx4 structure specific endonuclease domain. Human SLX4 is a Holliday junction resolvase subunit that binds multiple DNA repair/recombination endonucleases [10]. Its expression peaks at 48 hrs. Contig12361.0.g62 encodes a protein with a reverse transcriptase domain, although key catalytic residues are not conserved. Proteins with sequence identity are restricted to stichotrichs. Contig10642.0.g77 encodes a protein containing a YTH domain, which is now

recognized to bind m6A containing RNAs in diverse lineages [11, 12]. Thus, *O. trifallax* encodes a number of proteins with restricted phylogenetic conservation that appear to function in RNA and DNA processes whose expression is limited to macronuclear development, implying potential roles in DNA rearrangements.

1. Aravind L, Iyer LM, Anantharaman V: **The two faces of Alba: the evolutionary connection between proteins participating in chromatin structure and RNA metabolism**. *Genome Biol* 2003, **4**(10):R64.
2. Fetzer CP, Hogan DJ, Lipps HJ: **A PIWI homolog is one of the proteins expressed exclusively during macronuclear development in the ciliate Stylonychia lemnae**. *Nucleic Acids Res* 2002, **30**(20):4380-4386.
3. Adams J, Kelso R, Cooley L: **The kelch repeat superfamily of proteins: propellers of cell function**. *Trends Cell Biol* 2000, **10**(1):17-24.
4. Xue F, Cooley L: **kelch encodes a component of intercellular bridges in Drosophila egg chambers**. *Cell* 1993, **72**(5):681-693.
5. Kloetzel JA: **Compartmentalization of the developing macronucleus following conjugation in stylonychia and euplotes**. *J Cell Biol* 1970, **47**(2):395-407.
6. Murti KG: **Organization of genetic material in the macronucleus of hypotrichous ciliates**. *Handbook of genetics* 1976, **5**:113-137.
7. Murti KG: **Electron-microscopic observations on the macronuclear development of Stylonychia mytilus and Tetrahymena pyriformis (Ciliophora-Protozoa)**. *J Cell Sci* 1973, **13**(2):479-509.
8. Prescott DM, Murti KG, Bostock CJ: **Genetic apparatus of Stylonychia sp**. *Nature* 1973, **242**(5400):576, 597-600.
9. Swart EC, Bracht JR, Magrini V, Minx P, Chen X, Zhou Y, Khurana JS, Goldman AD, Nowacki M, Schotanus K *et al*: **The Oxytricha trifallax macronuclear genome: a complex eukaryotic genome with 16,000 tiny chromosomes**. *PLoS Biol* 2013, **11**(1):e1001473.
10. Fekairi S, Scaglione S, Chahwan C, Taylor ER, Tissier A, Coulon S, Dong MQ, Ruse C, Yates JR, 3rd, Russell P *et al*: **Human SLX4 is a Holliday junction resolvase subunit that binds multiple DNA repair/recombination endonucleases**. *Cell* 2009, **138**(1):78-89.
11. Dominissini D, Moshitch-Moshkovitz S, Schwartz S, Salmon-Divon M, Ungar L, Osenberg S, Cesarkas K, Jacob-Hirsch J, Amariglio N, Kupiec M *et al*: **Topology of the human and mouse m6A RNA methylomes revealed by m6A-seq**. *Nature* 2012, **485**(7397):201-206.
12. Schwartz S, Agarwala SD, Mumbach MR, Jovanovic M, Mertins P, Shishkin A, Tabach Y, Mikkelsen TS, Satija R, Ruvkun G *et al*: **High-resolution mapping reveals a conserved, widespread, dynamic mRNA methylation program in yeast meiosis**. *Cell* 2013, **155**(6):1409-1421.
