# Supplementary material for: Preferential expression of scores of functionally and evolutionarily diverse DNA and RNA-binding proteins during Oxytricha trifallax macronuclear development
Source: PLoS One. 2017 Feb 16;12(2):e0170870. doi: 10.1371/journal.pone.0170870 (PMC5312943; doi:10.1371/journal.pone.0170870)
Supplement: S3 Text — (PDF) [file pone.0170870.s018.pdf]

Further Characterization of Two Putative Regulatory Elements

The most significant downstream element is also associated with module 4 (motif 2); it is an AT(U) rich sequence with a terminal C that displays strong strand bias (log_2 = 2.8), occurs near the translation stop site (median 31 bases downstream vs 62 bases in background sequences, p = 0.0002, Figure 9C) and could potentially function to regulate mRNA stability. This motif *per se* is not significantly overrepresented in orthologous sequences from *S. histriomuscorum*, but a motif that overlaps by eight bases is the top scoring downstream motif from *de novo* discovery using HOMER (Figure 9C).

The top scoring upstream motif for module 3 (GTGAGTAAAT – motif 6, 18 mRNAs in module 3) is the reverse complement of the top scoring downstream motif (TTACTCAC – motif 15, 19 mRNAs in module 3). In nine cases the motif is present both upstream and downstream of the same coding sequence; thus half of these mRNAs contain the motif upstream and/or downstream. While preliminary, this work provides the first examples of potential cis-acting regulatory elements in *O. trifallax*.
